# A risk prediction model based on immune-inflammatory-nutritional indicators for predicting 28-day mortality in sepsis patients with acute respiratory distress syndrome

**DOI:** 10.3389/fnut.2026.1764044

**Published:** 2026-02-25

**Authors:** Qi Xin, Xingbo Dang, Gongliang Du, Yanlong Yang, Haitao Jing

**Affiliations:** Department of Emergency Surgery, Shaanxi Provincial People's Hospital, Xi'an, China

**Keywords:** 28-day mortality, acute respiratory distress syndrome, immune-inflammatory-nutritional indicators, nomogram, sepsis

## Abstract

**Background:**

Sepsis is a life-threatening condition often complicated by organ dysfunction and is associated with a high mortality rate. The dysregulation of immune response, inflammation, and nutritional status are critical factors contributing to its pathogenesis. This study aimed to develop a nomogram that integrates prognostic immune-inflammatory-nutritional indicators with other clinical information to predict 28-day mortality in sepsis patients with acute respiratory distress syndrome (ARDS).

**Methods:**

Clinical data from 635 adult sepsis patients with ARDS were obtained from Shaanxi Provincial People's Hospital and randomly divided into a training set (*n* = 477) and a validation set (*n* = 158). To identify predictors of 28-day mortality in sepsis patients with ARDS, univariate analysis, and least absolute shrinkage and selection operator (LASSO) regression were utilized. Subsequently, a multivariate logistic regression model was constructed to identify independent predictors. A nomogram was then developed by integrating the selected indicators. The model's performance was assessed with respect to discrimination, calibration, and clinical utility through the use of the area under the receiver operating characteristic curve (AUC), calibration plots, and decision curve analysis (DCA).

**Results:**

The independent predictors utilized for the construction of the nomogram included the albumin-alkaline phosphatase ratio (AAPR), albumin-bilirubin grade (ALBI), neutrophil–lymphocyte ratio (NLR), platelet-to-lymphocyte ratio (PLR), prognostic nutritional index (PNI), systemic immune-inflammation index (SII), and lactate-albumin ratio (LAR). Notably, the nomogram exhibited superior predictive performance, with an AUC of 0.873 in the training set and 0.837 in the validation set, as compared to the SOFA score, which showed an AUC of 0.689 in the training set and 0.684 in the validation set, for predicting 28-day mortality in sepsis patients with ARDS. The calibration plots demonstrated excellent consistency. DCA confirmed the model's clinical utility, showing a positive net benefit across a wide range of clinically relevant threshold probabilities (approximately 10% to 70%), which supports its potential to guide clinical decision-making.

**Conclusion:**

We have successfully developed and validated a robust nomogram that integrates seven readily accessible immune-inflammatory-nutritional indicators. This model serves as an individualized and precise tool for predicting the 28-day mortality risk in sepsis patients with acute respiratory distress syndrome (ARDS), thereby potentially enhancing early risk stratification and informing clinical decision-making.

## Introduction

Sepsis, characterized by life-threatening organ dysfunction due to a dysregulated host response to infection, is a leading cause of mortality in intensive care units (ICUs) globally, accounting for 25–30% of cases (1). ARDS, a severe and prevalent complication of sepsis, is marked by acute inflammatory lung injury, increased pulmonary vascular permeability, and hypoxemia. Notably, sepsis is a primary cause of ARDS, responsible for approximately 32% of ARDS cases (2). Research indicates that ARDS resulting from sepsis is more severe and associated with a poorer prognosis compared to ARDS arising from other etiologies (2, 3). The intersection of sepsis and ARDS culminates in a mortality rate exceeding 40%, posing a significant challenge in the field of critical care medicine (4).

The pathophysiology of sepsis-associated ARDS is characterized by a complex interplay of uncontrolled systemic inflammation, immune cell dysfunction, and metabolic alterations (5). Consequently, factors such as the host's immune status, the intensity of the inflammatory response, and the underlying nutritional reserves emerge as critical determinants of clinical outcomes. In this context, composite biomarkers that reflect these interconnected pathways have attracted increasing attention. Indicators such as the neutrophil-to-lymphocyte ratio (NLR), the systemic immune-inflammation index (SII), and the prognostic nutritional index (PNI) have demonstrated potential in predicting outcomes in various critical illnesses, including sepsis and ARDS (6–11). However, these biomarkers are frequently assessed in isolation, and there remains a lack of a comprehensive model that integrates multiple dimensions of the host response. Although the Sequential Organ Failure Assessment (SOFA) score is widely employed for evaluating organ dysfunction, it may not fully encapsulate the prognostic information inherent in the immune-inflammatory-nutritional axis (1). Thus, there is an urgent need for the development of a more specialized and accurate predictive tool.

Recently, a prognostic scoring system that integrates immune, inflammatory, and nutritional parameters has demonstrated substantial clinical utility in predicting outcomes in cancer and other critical illnesses (12–15). Building on this concept, our study aimed to adapt and apply this multidimensional approach to sepsis-associated ARDS. We selected readily available laboratory parameters that reflect the intertwined pathways of inflammation, immune stress, and metabolic/nutritional reserve depletion, acknowledging that in acute sepsis, traditional nutritional markers like albumin serve as proxies for this complex interplay rather than standalone nutritional assessments. We hypothesized that a nomogram model, which incorporates immune-inflammatory-nutritional indicators alongside clinical variables, could improve the early prediction of 28-day mortality in this high-risk population. Consequently, the objective of this study was to develop and validate such a nomogram to facilitate early prognostic assessment and enable tailored clinical management.

## Materials and methods

### Data source

This retrospective cohort study analyzed data from the Shaanxi Provincial People's Hospital, spanning the period from January 2020 to October 2025. The study protocol, including the collection of clinical data, was approved by the Medical Research Ethics Committee of the Shaanxi Provincial People's Hospital (Approval No: 2025R082).

### Study population

The patients included in this study were adult ICU patients who met both the Sepsis-3 criteria and the Berlin definition of ARDS. The diagnosis of sepsis was based on the Sepsis-3 criteria, requiring clear evidence of infection (based on clinical, microbiological, or imaging assessment), and a sequential organ failure assessment (SOFA) score increasing by ≥ 2 points compared to the baseline within 24 h of admission (1). The diagnosis of ARDS strictly follows the Berlin definition, which stipulates that patients must develop new or exacerbated respiratory symptoms within 1 week of the onset of sepsis. Chest imaging should show bilateral pulmonary infiltrates, and the oxygenation index (PaO_2_/FiO_2_) should meet the criteria for hypoxemia when the positive end-expiratory pressure (PEEP) is ≥ 5 cmH_2_O. At the same time, exclusion of heart failure or excessive fluid load as the main cause is required (16). All diagnoses were independently reviewed by two senior critical care physicians. In case of any disagreement, a third expert would make the final decision.

This study aims to develop a prognostic prediction model for the clinical syndrome of “simultaneous sepsis and ARDS,” rather than distinguishing whether ARDS is caused by extrapulmonary sepsis or pneumonia. Therefore, the inclusion criteria do not strictly require the initial site of infection to be differentiated (either within the lungs or outside the lungs) but rather consider both as a clinical entity with common pathophysiological characteristics. Nevertheless, we still recorded all patients' suspected infection foci (as baseline variables) and explored the impact of different infection sites on the model's predictive performance in the sensitivity analysis.

The exclusion criteria were: (1) patients younger than 18 years; (2) an ICU stay of less than 24 h; (3) patients with chronic liver diseases (including liver cirrhosis, chronic hepatitis, etc.); (4) patients with bone marrow suppression, active malignancies, or those receiving chemotherapy/antiplatelet therapy, (5) patients with pregnant or lactating, and (6) incomplete key data necessary for analysis.

### Data extraction and predictor variables

The following variables were collected within the first 24 h of admission to the intensive care unit (ICU): (1) Demographic and Vital Sign Data: age, gender, body temperature, heart rate, respiratory rate, and mean arterial pressure; (2) Comorbidities: hypertension, diabetes, respiratory failure, septic shock, acute kidney injury (AKI), atrial fibrillation, and chronic kidney disease; (3) Laboratory Parameters: aspartate aminotransferase, alanine aminotransferase, glucose, white blood cell count, procalcitonin, C-reactive protein, uric acid, total cholesterol, cystatin C, blood urea nitrogen, and creatinine; (4) Nine Candidate Immune-Inflammatory-Nutritional Indicators: albumin-alkaline phosphatase ratio (AAPR), albumin-bilirubin (ALBI) grade, NLR, platelet-to-lymphocyte ratio (PLR), lymphocyte-to-monocyte ratio (LMR), PNI, SII, and lactate-albumin ratio (LAR); (5) Clinical Scores: SOFA score. The formulas for the calculations are presented as follows: the AAPR is determined by dividing albumin (g/L) by alkaline phosphatase (ALP) (IU/L); the PNI is calculated by adding albumin to five times the lymphocyte count; and the SII is computed as platelet count multiplied by neutrophil count, divided by lymphocyte count. The ALBI grade is classified according to the following thresholds: ≤ −2.60 for ALBI grade 1, > −2.60 to ≤ −1.39 for ALBI grade 2, and ≥ −1.39 for ALBI grade 3, as previously established in the literature.

### The primary endpoint

This study selected the 28-day all-cause mortality rate as the primary endpoint. The following considerations were taken into account: (1) It is widely accepted as a standard time point for evaluating the short-term prognosis of critical conditions (especially sepsis and ARDS), facilitating comparisons with a large number of existing studies; (2) Compared with in-hospital mortality rate, the 28-day mortality rate can more comprehensively capture early death events, avoiding bias caused by differences in discharge policies among different medical institutions; (3) It is located at the period with the highest risk of sepsis-related death, providing direct clinical guidance value for early risk stratification and intervention.

For patients who were discharged before the end of the study period (October 2025) and whose 28-day outcomes were not recorded in the hospital, we conducted outcome confirmation through the following structured process to ensure the completeness of the data: (1) Electronic medical record system query: Firstly, in the integrated electronic medical record system of the hospital, check whether the patient was readmitted within 28 days after discharge for any reason, and record the survival status of the patient during this hospitalization. (2) Telephone follow-up: For patients for whom no record of readmission was found, the research team followed a standardized follow-up procedure and attempted to contact the patient or their designated immediate family member by phone for a follow-up call. The main objective is to determine whether the patient is still alive on the 28th day after being admitted to the ICU.

### Data completeness

Following the application of the exclusion criteria (in particular, exclusion of patients with incomplete key data), the final analytical cohort of 635 patients constituted a complete-case dataset. A systematic verification confirmed that there were no missing values for any of the demographic, clinical, laboratory, or immune-inflammatory-nutritional predictor variables listed in the Data Extraction section among these 635 patients. Consequently, no imputation methods were required or applied.

### Sample size consideration and overfitting assessment

This was a retrospective, single-center study utilizing all available eligible data from the defined study period to maximize the sample size for model development. While a formal a priori sample size calculation for prediction model development is complex and depends on the anticipated outcome incidence and predictor effects, we followed established guidelines to ensure robustness. A key metric to guard against overfitting in logistic regression is the Events Per Variable (EPV). It is recommended that the EPV should be at least 10 to 20 to ensure reliable and generalizable parameter estimates (17). Our final model incorporated 7 predictors. In the training set (*n* = 477), we observed 226 events of 28-day mortality. This yields an EPV of 32.3 (226/7), which comfortably exceeds the recommended thresholds, indicating a very low risk of overfitting and affirming the adequacy of our sample size for the performed multivariable modeling.

### Statistical analysis

The study cohort was randomly divided, using a fixed random seed (Seed = 20,231,001), into a training set (75%) for model development and an independent hold-out validation set (25%) for model evaluation. Continuous variables adhering to a normal distribution were reported as mean ± standard deviation, whereas non-normally distributed variables were presented as median (interquartile range). Categorical variables were represented as percentages. The model development process comprised three steps: (1) Univariate analysis: All potential indicators were analyzed using univariate logistic regression to identify those preliminarily associated with 28-day mortality (*P* < 0.05); (2) Handling of Continuous Predictors: Continuous predictors were analyzed on their original scale. The linearity assumption for each variable against the log-odds of 28-day mortality was assessed using smooth calibration plots and Martingale residual plots. Where non-linearity was evident, restricted cubic splines (RCS) were considered. However, for the selected composite indicators—which are typically interpreted monotonically—and based on our diagnostic checks, a linear form in the logit was judged appropriate to preserve simplicity and clinical interpretability. No continuous predictors were arbitrarily categorized. (3) LASSO: To mitigate overfitting and multicollinearity, the indicators significantly associated in the univariate analysis were included in a LASSO logistic regression model; (4) Nomogram construction: A multivariate logistic regression was performed using the features selected from the LASSO analysis to identify independent predictors, followed by the construction of a nomogram. The model's performance was evaluated in both the training and validation sets. Discrimination was assessed using the area under the receiver operating characteristic curve (AUC). Calibration was evaluated through the use of calibration plots incorporating a non-parametric smoothing technique. The clinical utility was quantified via decision curve analysis (DCA), which calculates the net benefit across a range of threshold probabilities.

Quantitative calibration was assessed by calculating the calibration slope and calibration intercept along with their 95% confidence intervals (obtained via bootstrapping with 1,000 replicates). A calibration slope of 1 and an intercept of 0 indicates perfect calibration. While the Hosmer-Lemeshow (H-L) goodness-of-fit test is commonly reported, it has known limitations, including sensitivity to sample size and arbitrary grouping of predicted probabilities. Therefore, we prioritized the use of the calibration slope and intercept, along with visual calibration plots, as more informative and robust measures of calibration performance.

All statistical analyses were conducted utilizing R software (version 4.1.3). A two-sided *P*-value of less than 0.05 was regarded as indicative of statistical significance.

## Result

### Basic characteristics

**Figure 1:**
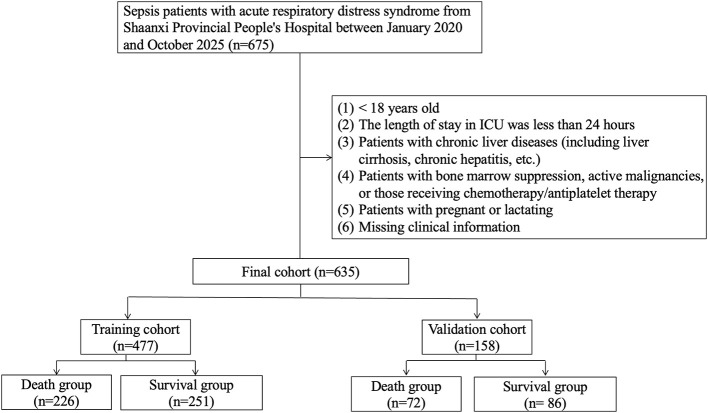
The flowchart of patient selection.

The study comprised 635 eligible participants, among whom 298 individuals (46.9%) experienced 28-day mortality, as depicted in Figure 1 and detailed in Table 1. The cohort was randomly divided into a training group (75%, *n* = 477) and a validation group (25%, *n* = 158). Table 1 presents comparable demographic characteristics, vital signs, comorbidities, laboratory examinations, immune-inflammatory-nutritional indicators, and outcome data for both the training and validation groups. The median age of participants in the training group was 65 years, with a gender distribution of 269 males (59.5%) and 208 females (40.5%). Similarly, the validation group had a median age of 63 years, comprising 86 males (60.4%) and 72 females (45.6%).

**Table 1:** Baseline characteristics of sepsis patient with acute respiratory distress syndrome.

**Variable**	**Total (*n* = 635)**	**Training (*n* = 477)**	**Validation (*n* = 158)**	***p*-value**
Morality, *n* (%)				0.762
No	337 (53.1)	251 (52.6)	86 (54.4)	
Yes	298 (46.9)	226 (47.4)	72 (45.6)	
Age	64 (52, 75)	65 (53, 76)	63 (49, 72)	0.193
Gender, *n* (%)				0.735
Female	280 (44.1)	208 (40.5)	72 (39.6)	
Male	355 (55.9)	269 (59.5)	86 (60.4)	
**Vital signs**			
T (°*C*)	36.6 (36.3, 37.0)	36.6 (36.4, 37.0)	36.5 (36.2, 37.2)	0.157
RR (bpm)	21 (19, 27)	21 (19, 27)	21 (19, 27)	0.975
HR (bpm)	98 (81, 116)	97 (80, 115)	98 (83, 117)	0.550
MAP (mmHg)	88.7 (76.7, 99.3)	87.3 (76.5, 98.8)	92 (77, 102)	0.057
**Comorbidities**			
Hypertension, *n* (%)				0.516
No	386 (60.7)	286 (60.0)	100 (63.3)	
Yes	249 (39.2)	191 (40.0)	58 (36.7)	
Diabetes, *n* (%)				0.965
No	453 (71.3)	341 (71.5)	112 (70.9)	
Yes	182 (28.7)	136 (28.5)	46 (29.1)	
Respiratory failure, *n* (%)				0.801
No	314 (49.4)	234 (49.1)	80 (50.6)	
Yes	321 (50.6)	243 (50.9)	78 (49.4)	
Septic shock, *n* (%)				0.799
No	326 (51.3)	243 (50.9)	83 (52.5)	
Yes	309 (48.7)	234 (49.1)	75 (47.5)	
AKI, *n* (%)				1
No	321 (50.6)	241 (50.5)	80 (50.6)	
Yes	314 (49.4)	236 (49.5)	78 (49.4)	
Atrial fibrillation, *n* (%)				0.567
No	548 (86.3)	409 (85.7)	139 (88.0)	
Yes	87 (13.7)	68 (14.3)	19 (12.0)	
CKD, *n* (%)				0.794
No	561 (88.3)	420 (88.1)	141 (89.2)	
Yes	74 (11.7)	57 (11.9)	17 (10.8)	
**Laboratory test**			
AST (U/L)	38.0 (25.0, 74.0)	38.0 (24.5, 72.0)	38.0 (25.0, 77.3)	0.568
ALT (U/L)	31.0 (18.0, 59.0)	31.0 (18.0, 54.0)	31.0 (18.8, 77.3)	0.327
Glucose (mmol/L)	7.86 (5.79, 11.12)	7.73 (5.68, 11.08)	8.06 (5.91, 11.32)	0.551
WBC ( × 10^9^/L)	9.55 (6.77, 15.99)	9.52 (6.54, 16.15)	9.64 (7.02, 15.90)	0.862
PCT (ng/ml)	2.26 (0.61, 13.00)	2.26 (0.59, 14.20)	2.22 (0.74, 10.28)	0.680
CRP (mg/L)	101 (37, 188)	101 (36, 187)	98 (37, 199)	0.821
UA (μmol/L)	305 (189, 423)	300 (183, 416)	317 (207, 446)	0.115
TC (mmol/L)	2.79 (2.03, 3.69)	2.79 (2.04, 3.78)	2.79 (2.00, 3.52)	0.400
Cystatin-C (mg/L)	1.50 (1.03, 2.63)	1.51 (1.04, 2.62)	1.44 (0.98, 2.65)	0.534
BUN (mmol/L)	11.0 (6.9,18.8)	10.9 (6.7, 18.2)	11.4 (0.98, 2.65)	0.254
Cr (μmol/L)	94.0 (57.0, 206.0)	94.0 (56.0, 197.5)	97.5 (60.0, 224.8)	0.333
CRRT, *n* (%)				0.421
No	377 (59.4)	288 (60.4)	89 (56.3)	
Yes	258 (40.6)	189 (39.6)	69 (43.7)	
SOFA	7 (7,10)	7 (7, 9)	7 (7, 10)	0.052
Hospital LOS (days)	14 (8, 24)	14 (8, 25)	15 (8, 24)	0.724
**Immune-inflammatory-nutritional indicators**			
AAPR	0.28 (0.17, 0.42)	0.29 (0.18, 0.42)	0.27 (0.16, 0.42)	0.621
ALBI grade				0.977
Grade 1	30 (4.7)	23 (4.8)	7 (4.4)	
Grade 2	278 (43.8)	209 (43.8)	69 (43.7)	
Grade 3	327 (51.5)	245 (51.4)	82 (51.9)	
NLR	16.9 (7.3, 34.5)	17.2 (7.4, 34.5)	15.3 (6.9, 34.1)	0.366
PLR	215 (113, 396)	222 (116, 419)	190 (107, 347)	0.131
LMR	1.38 (0.54, 3.10)	1.37 (0.52, 3.02)	1.45 (0.70, 3.38)	0.304
PNI	32.2 (28.0, 36.5)	32.3 (28.1, 36.5)	32.1 (27.7, 36.3)	0.555
SII	1982 (923, 4703)	2062 (925, 4806)	1701 (804, 4095)	0.337
LAR	0.07 (0.05,0.12)	0.07 (0.05, 0.12)	0.07 (0.05, 0.13)	0.867

T, body temperature; HR, heart rate; RR, respiratory rate; MAP, mean arterial pressure; AKI, acute kidney injury; CKD, chronic kidney disease; AST, aspartate transaminase; ALT, alanine aminotransferase; WBC, white blood cell; PCT, procalcitonin; CRP, C-reactive protein; UA, uric acid; TC, total cholesterol; CRRT, continuous renal replacement therapy; BUN, blood urea nitrogen; Cr, creatinine; SOFA, sequential organ failure assessment; ICU, intensive care unit; LOS, Length of stay; AAPR, album-alkaline phosphatase ratio; ALBI, albumin-bilirubin; NLR, neutrophil-lymphocyte ratio; PLR, platelet-to-lymphocyte ratio; LMR, lymphocyte-to-monocyte ratio; PNI, prognostic nutritional index; SII, systemic immune-inflammation index; LAR, lactate-albumin ratio.

### Univariate analysis results

All demographic and clinical data were accessible within 24 h of admission. Univariate analyses, as presented in Table 2, indicated that the levels of WBC (1.066, 95% CI: 1.036–1.096), Cystatin C (1.129, 95% CI: 1.002–1.271), ALBI (4.024, 95% CI: 2.810–5.763), NLR (1.022, 95% CI: 1.014–1.031), and SII (1.000, 95% CI: 1.000–1.000), as well as LAR (1256205.6, 95% CI: 23135.7–68208580.1), were significantly elevated in the Death group compared to the Survival group. Conversely, AAPR (0.020, 95% CI: 0.006–0.067), PLR (0.999, 95% CI: 0.999–1.000), LMR (0.987, 95% CI: 0.962–1.013), and PNI (0.902, 95% CI: 0.875–0.931) were significantly lower in the Death group.

**Table 2:** Univariate analysis of predictive variables of the 28-day mortality in the training cohort.

**Variables**	**OR**	**95% CI**	***p*-value**
Age (years)	1.002	0.991–1.013	0.722
Gender	0.920	0.640–1.321	0.650
**Vital signs**			
T (°*C*)	0.902	0.734–1.107	0.323
RR (bpm)	0.983	0.960–1.007	0.155
HR (bpm)	1.001	0.994–1.008	0.780
MAP (mmHg)	0.996	0.985–1.007	0.454
**Comorbidities**			
Hypertension	0.885	0.613–1.277	0.513
Diabetes	1.158	0.778–1.724	0.469
Respiratory failure	1.304	0.909–1.869	0.149
Septic shock	1.315	0.917–1.886	0.136
AKI	1.318	0.919–1.889	0.134
Atrial fibrillation	1.211	0.724–2.023	0.466
CKD	0.851	0.488–1.486	0.571
**Laboratory test**			
AST (U/L)	1.000	0.999–1.001	0.843
ALT (U/L)	1.000	0.999–1.001	0.875
Glucose (mmol/L)	1.012	0.980–1.046	0.463
WBC (×10^9^/L)	1.066	1.036–1.096	< 0.001
PCT (ng/ml)	1.003	0.997–1.010	0.278
CRP (mg/L)	1.001	0.999–1.003	0.185
UA (μmol/L)	1.000	0.999–1.001	0.990
TC (mmol/L)	1.000	0.876–1.142	0.998
Cystatin-C (mg/L)	1.129	1.002–1.271	0.046
BUN (mmol/L)	1.014	0.996–1.033	0.136
Cr (μmol/L)	1.001	1.000–1.002	0.081
CRRT, *n* (%)	1.300	0.900–1.877	0.163
SOFA	1.404	1.278–1.541	< 0.001
Hospital LOS (days)	0.946	0.929–0.962	< 0.001
AAPR	0.020	0.006–0.067	< 0.001
ALBI	4.024	2.810–5.763	< 0.001
NLR	1.022	1.014–1.031	< 0.001
PLR	0.999	0.999–1.000	0.017
LMR	0.987	0.962–1.013	0.317
PNI	0.902	0.875–0.931	< 0.001
SII	1.000	1.000–1.000	0.003
LAR	1256205.6	23135.7–68208580.1	< 0.001

T, body temperature; HR, heart rate; RR, respiratory rate; MAP, mean arterial pressure; AKI, acute kidney injury; CKD, chronic kidney disease; AST, aspartate transaminase; ALT, alanine aminotransferase; WBC, white blood cell; PCT, procalcitonin; CRP, C-reactive protein; UA, uric acid; TC, total cholesterol; CRRT, continuous renal replacement therapy; BUN, blood urea nitrogen; Cr, creatinine; SOFA, sequential organ failure assessment; ICU, intensive care unit; LOS, Length of stay; AAPR, album-alkaline phosphatase ratio; ALBI, albumin-bilirubin; NLR, neutrophil-lymphocyte ratio; PLR, platelet-to-lymphocyte ratio; LMR, lymphocyte-to-monocyte ratio; PNI, prognostic nutritional index; SII, systemic immune-inflammation index; LAR, lactate-albumin ratio.

### Development of nomogram

**Figure 2:**
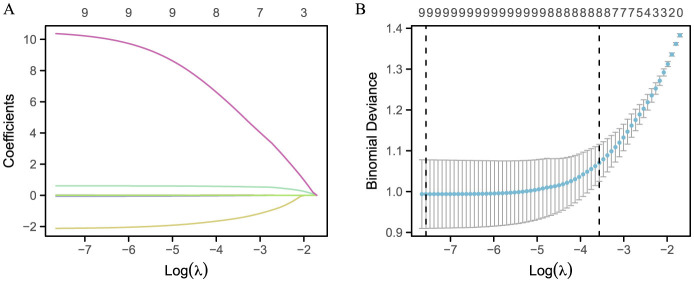
Selection of clinical features using LASSO. **(A)** The plot illustrates the LASSO coefficient profiles for 10 features, with the log-transformed lambda sequence plotted against the coefficient profiles. Eight features exhibited non-zero coefficients at the optimal lambda value. **(B)** Ten-fold cross-validation was employed for tuning the parameters of the LASSO model. The binomial deviance curve is depicted with respect to the log-transformed lambda. Dotted vertical lines represent the optimal values determined by the minimum criteria and its one standard error, adhering to the 1-SE criterion.

**Figure 3:**
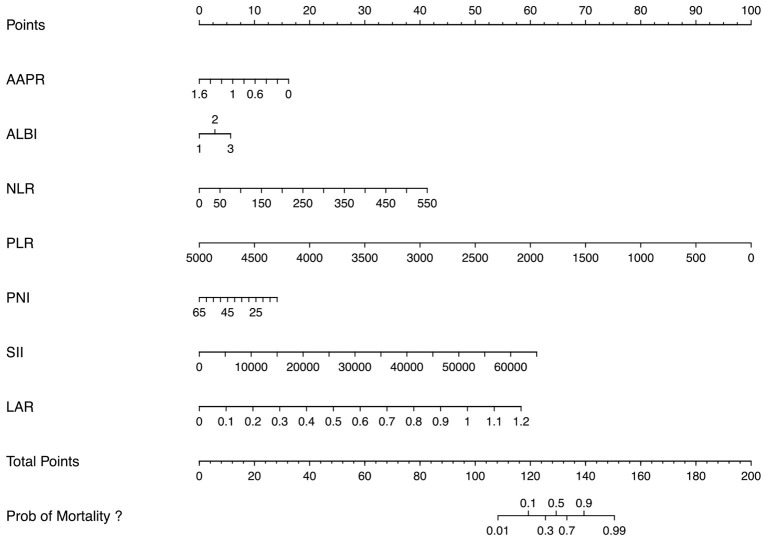
Nomogram model for predicting 28-day mortality in sepsis patients with ARDS. AAPR, album-alkaline phosphatase ratio; ALBI, albumin-bilirubin; NLR, neutrophil-lymphocyte ratio; PLR, platelet-to-lymphocyte ratio; PNI, prognostic nutritional index; SII, systemic immune-inflammation index; LAR, lactate-albumin ratio.

A 10-fold cross-validation of the initial input LASSO regression method was performed to mitigate collinearity among the relevant indicators, facilitating the identification of mortality predictors (Figure 2). Ultimately, eight variables were selected as optimal based on the optimal lambda value. The model was formulated as follows: −1.125558019 + 015333867 ^*^ WBC - 1.487836505 ^*^ AAPR + 0.563129312 ^*^ ALBI + 0.009042757 ^*^ NLR - 0.000925553 ^*^ PLR - 0.018924139 ^*^ PNI + 2.38664 ^*^ 10^−05^^*^ SII + 5.52519249 ^*^ LAR. A multivariate logistic regression analysis of the training set, employing these variables, revealed that AAPR, ALBI, NLR, PLR, PNI, SII, and LAR were independent risk factors for the development of 28-day mortality in sepsis patients with ARDS (Table 3). Based on these variables, a nomogram was developed to predict 28-day mortality in sepsis patients with ARDS (Figure 3).

**Table 3:** Multivariate logistic regression analysis of independent predictors of the 28-day mortality in the training cohort.

**Variables**	**β**	**SE**	**Wald**	***p*-value**	**OR (95% Cl)**
AAPR	−2.203	0.745	8.749	0.003	0.110 (0.026–0.476)
ALBI	0.619	0.277	5.003	0.025	1.856 (1.080–3.194)
NLR	0.016	0.005	10.612	0.001	1.016 (1.007–1.027)
PLR	−0.004	0.001	9.453	< 0.001	0.996 (0.994–0.997)
PNI	−0.056	0.025	5.137	0.023	0.946 (0.9012–0.992)
SII	0.000	0.000	18.535	< 0.001	1.000 (1.000–1.000)
LAR	10.585	2.353	20.235	< 0.001	39549 (393–3982433)
Constant	0.144	1.369	0.011	0.916	1.155

AAPR, album-alkaline phosphatase ratio; ALBI, albumin-bilirubin; NLR, neutrophil-lymphocyte ratio; PLR, platelet-to-lymphocyte ratio; PNI, prognostic nutritional index; SII, systemic immune-inflammation index; LAR, lactate-albumin ratio.

### Verification of nomogram

**Figure 4:**
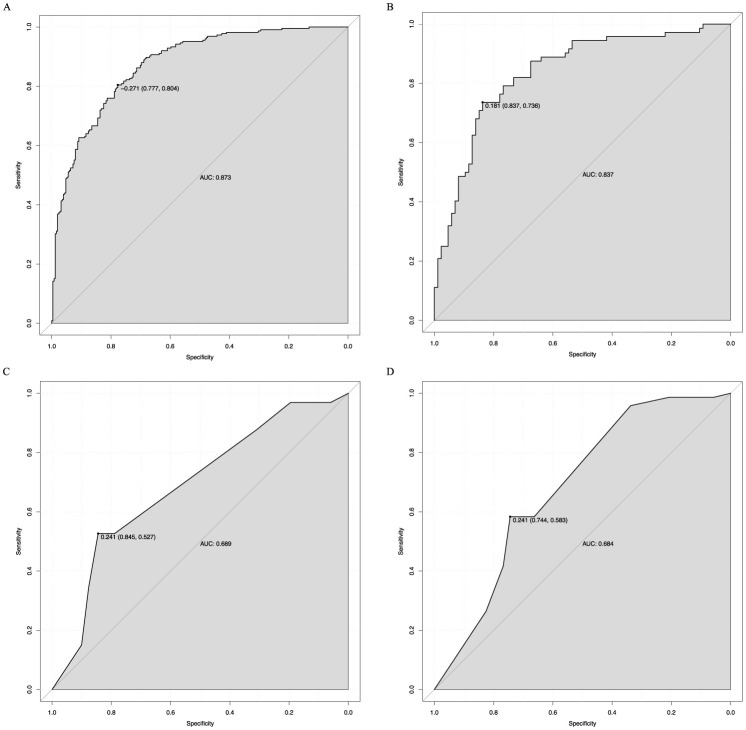
The ROC curve of the nomogram for predicting 28-day mortality in sepsis patients with ARDS. The AUC of the nomogram for the prediction of 28-day mortality in sepsis patients with ARDS was 0.873 in the training set **(A)** and 0.837 in the validation set **(B)**. The AUC of SOFA for the prediction of 28-day mortality in sepsis patients with ARDS was 0.689 in the training set **(C)** and 0.684 in the validation set **(D)**. ROC, receiver operating characteristic; AUC, area under the receiver operating characteristics curve; SOFA, sequential organ failure assessment.

**Figure 5:**
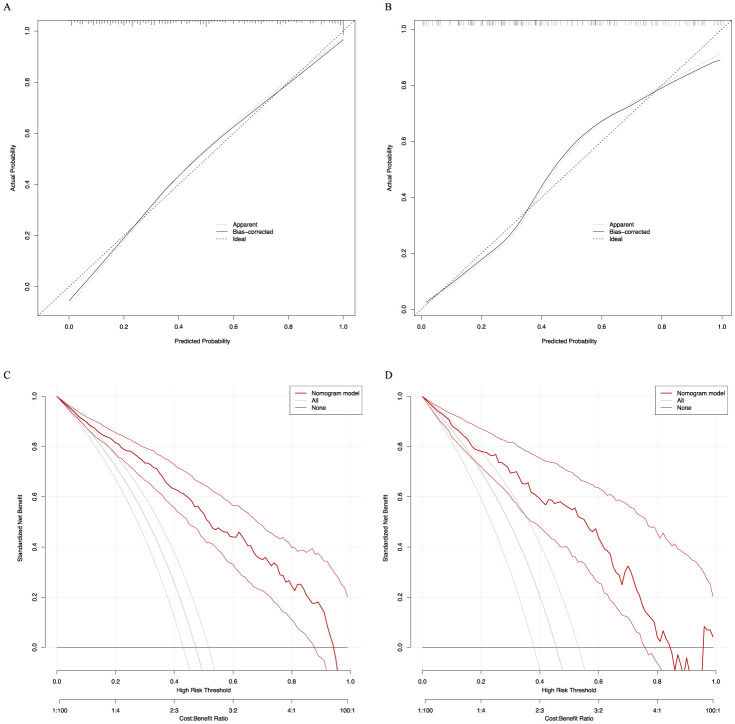
Calibration curves and DCA of the nomogram for predicting 28-day mortality. Calibration curves of the predicted nomogram in the training set **(A)** and validation set **(B)**; DCA of the nomogram in the training set **(C)** and the validation set **(D)**. DCA, Decision curve analysis.

The analysis of the ROC curve indicated that the nomogram exhibited a strong capacity to predict 28-day mortality in sepsis patients with ARDS within both the training (AUC = 0.873) and validation (AUC = 0.837) cohorts, as depicted in Figure 4. Furthermore, the simplified prediction model demonstrated superior predictive performance compared to the SOFA score in both the training (AUC = 0.689) and validation (AUC = 0.684) cohorts (Figure 4). The calibration plots demonstrated excellent agreement between predicted and observed risks (Figure 5). Quantitatively, the calibration slope was 1.01 (95% CI: 0.97–1.05) with an intercept of 0.02 (95% CI: −0.03–0.07) in the training set. In the validation set, the slope was 0.95 (95% CI: 0.90–1.00) and the intercept was −0.03 (95% CI: −0.10–0.04). These metrics indicate nearly ideal calibration in the training set and excellent calibration with only minimal shrinkage in the validation set, suggesting negligible overfitting.

DCA further quantified the clinical utility of the nomogram (Figures 5C, D). Compared to the “treat-all” or “treat-none” strategies, the use of our nomogram to guide intervention decisions provided a superior net benefit across a clinically practical range of threshold probabilities from approximately 10% to 70% in both the training and validation sets. This range encompasses the gray zone where clinical decision-making is often most uncertain. For example, at a threshold probability of 30% (meaning a clinician would consider an intervention if the patient's predicted mortality risk is ≥30%), the net benefit of using the nomogram was approximately 0.08 in the validation set, indicating that for every 100 patients, using the model would lead to 8 more beneficial interventions without increasing unnecessary interventions, compared to not using any model.

## Discussion

In this retrospective study, we developed and validated an innovative prognostic nomogram that integrates seven immune-inflammatory-nutritional indicators to predict 28-day mortality in sepsis patients with acute respiratory distress syndrome (ARDS). The model exhibited exceptional discriminatory power and calibration, significantly surpassing the conventional Sequential Organ Failure Assessment (SOFA) score. This model is specifically designed for this high-risk population, comprehensively incorporating biomarkers from the immune, inflammatory, and nutritional domains. The strength of our model lies in its multidimensional nature, which reflects the complex pathophysiology of sepsis-associated ARDS.

The seven selected biomarkers—AAPR, ALBI, NLR, PLR, PNI, SII, and LAR—collectively provide a detailed depiction of the patient's pathophysiological state. For example, a low AAPR, indicative of hypoalbuminemia and/or elevated alkaline phosphatase, has been associated with increased mortality in critically ill patients, potentially signaling hepatic dysfunction and systemic inflammation (18, 19). Alkaline Phosphatase (ALP) is an enzyme of considerable importance, playing a pivotal role in bone mineralization and hepatic function. Recent research has elucidated that ALP is capable of influencing intercellular signal transduction and modulating inflammatory responses (20, 21). The occurrence of hypoalbuminemia, an essential component of the AAPR and the PNI, may indicate malnutrition, chronic inflammation, or compromised synthetic function, all of which are commonly observed in critically ill patients (22). The ALBI grade, initially developed for assessing liver cancer, demonstrated significant prognostic utility within our cohort of patients with sepsis-associated ARDS (23). This finding implies that the liver's synthetic function, as indicated by levels of albumin and bilirubin, is a critical factor influencing survival in critical illness, extending beyond primary hepatic disorders. This may underscore the liver's integral role in modulating systemic inflammation and synthesizing acute-phase proteins.

The NLR and SII are well-established indicators of systemic inflammation and immune stress (24, 25). Elevated levels of these markers indicate a predominance of neutrophils and relative lymphopenia, conditions linked to endothelial damage and immune suppression in sepsis (26–28). An increase in neutrophil count is correlated with more severe vascular endothelial damage and an intensified immune response in sepsis patients (28). Conversely, a reduction in lymphocyte count is associated with immunosuppression and impaired pathogen clearance, characteristic of sepsis-induced immunoparalysis (29, 30). During sepsis, endotoxins stimulate monocytes, neutrophils, macrophages, T cells, and other immune cells to release substantial quantities of endogenous inflammatory mediators through a complex immune network response, resulting in the amplification and dysregulation of the inflammatory response. The PLR, another composite index involving platelets and lymphocytes, may reflect the interaction between inflammation and thrombosis. This is particularly pertinent in ARDS, where microvascular thrombosis contributes to organ dysfunction (8, 31, 32). Our study identified the NLR, SII, and PLR as robust prognostic indicators for predicting 28-day mortality in sepsis patients with ARDS.

A notable advantage of our model is the incorporation of nutritional and metabolic markers, which distinguishes it from models such as the SOFA. The PNI, calculated from albumin levels and lymphocyte count, serves as an integrated marker of nutritional status and immune competence. A low PNI is indicative of malnutrition and compromised immunity, conditions known to exacerbate outcomes in critical illnesses (33, 34). Similarly, the LAR combines a marker of cellular stress and anaerobic metabolism (lactate) with a marker of nutritional status and anti-inflammatory capacity (albumin). An elevated LAR indicates tissue hypoperfusion and a catabolic state, offering a potent prognostic signal, as supported by recent studies in septic patients (35, 36). One study demonstrated that LAR possesses significant prognostic value in newly diagnosed sepsis patients, particularly at admission and 1 week thereafter, with higher LAR levels correlating with adverse outcomes (35). Furthermore, the LAR has demonstrated a superior predictive capability for sepsis-related acute kidney injury (S-AKI), surpassing traditional disease severity scores (36). Elevated lactate levels in the context of sepsis may indicate tissue hypoperfusion and inadequate oxygenation, while hypoalbuminemia may be indicative of malnutrition or chronic inflammation. Empirical studies have established a strong correlation between elevated lactate levels and increased mortality rates in sepsis patients, particularly when lactate concentrations exceed 4 mmol/L, at which point the correlation becomes even more pronounced (37). Therefore, the LAR emerges as a valuable composite biomarker for evaluating the severity and prognosis of critical illness.

The nomogram developed in this study achieved AUC values of 0.873 and 0.837 in the training set and validation set respectively. Its discrimination was significantly superior to the SOFA score used as the benchmark in this study (AUC: 0.689–0.684). This performance is comparable to, and even superior to, other models reported in recent literature. Unlike many traditional models (such as APACHE II and SAPS II) that mainly rely on vital signs, age, and organ function scores, nor are they similar to some models that only contain a single inflammatory indicator (such as NLR or PCT), the uniqueness of this model lies in its systematic integration of multiple dimensions of easily accessible immune, inflammatory, and nutritional metabolism-related biomarkers. Our model addresses this limitation by providing a more comprehensive evaluation of the host's biological condition, thereby serving as a potential tool for earlier and more personalized intervention. For instance, a patient identified as high-risk by our nomogram might benefit from more aggressive immunomodulatory therapy, targeted nutritional support, or closer monitoring, even if their SOFA score indicates only moderate elevation. Our model's inclusion of albumin-derived ratios (e.g., PNI, LAR) captures aspects of the catabolic state, hepatic synthesis, and inflammatory burden, which are crucial surrogates for the metabolic and nutritional stress in early sepsis, even though they are not pure nutritional markers.

## Example of nomogram application

To illustrate the practical use of the nomogram (Figure 3), consider a hypothetical sepsis patient with ARDS with the following admission profile: AAPR = 0.6, ALBI grade = 3, NLR = 150, PLR = 1,500, PNI = 45, SII = 10,000, and LAR =0.4. Using the nomogram, points are assigned for each variable: approximately 10 points for AAPR, 6 for ALBI grade, 10 for NLR, 70 for PLR, 5 for PNI, 10 for SII, and 20 for LAR. The total points sum to approximately 131. By projecting this total points value to the bottom risk axis, the estimated 28-day mortality probability for this patient is approximately 70%.

## Limitations

Several limitations of this study warrant acknowledgment. Firstly, the retrospective and single-center design may introduce selection bias and restrict the generalizability of the findings. Despite robust performance demonstrated through internal validation, external validation in prospective, multi-center cohorts is crucial prior to clinical application. Secondly, the model is limited by the variables available for analysis. Unmeasured confounders, such as specific microbiological data, details of antimicrobial therapy, or long-term functional status, may influence the outcomes. Specifically, the “nutritional” indicators in our model (primarily albumin-based) are influenced by acute-phase responses and fluid shifts in sepsis, and do not represent comprehensive nutritional assessment. The absence of dedicated malnutrition screening tools or detailed anthropometric/dietary intake data is a limitation of this retrospective study. Thirdly, the study did not account for the dynamic nature of biomarkers, as only values from the first 24 h were utilized. Moreover, and of critical importance, our retrospective design limited our ability to capture and adjust for variations in key immunomodulatory treatments. Finally, we were unable to account for the potential confounding effect of variations in nutritional support practices (e.g., timing, route, composition) received by the patients after ICU admission. The lack of standardized data on Medical Nutrition Therapy or immunomodulatory formulas limits our ability to assess how such interventions might interact with the baseline biomarkers in our model or influence outcomes. Future research incorporating trajectory data could potentially enhance predictive accuracy. Moreover, to strengthen the generalizability and clinical applicability of the model, the authors should seek external validation using data from different centers or populations in future studies.

## Methodological considerations

Regarding model development, we employed LASSO regression for variable selection to mitigate multicollinearity and overfitting. While this approach is data-driven and efficient, it carries the inherent risk of excluding weak but potentially clinically informative predictors if their effect sizes fall below the penalization threshold. To address this, we set the LASSO tuning parameter (λ) using 10-fold cross-validation based on the “1 standard error” (1-SE) rule (as shown in Figure 2B), which favors a more parsimonious and stable model over the absolute best fit. Furthermore, all candidate variables were pre-screened based on biological plausibility and existing literature in sepsis and ARDS, ensuring that the selection pool was clinically grounded. Nevertheless, the final set of predictors should be interpreted as a robust, parsimonious combination for prediction, rather than an exhaustive causal list.

## Conclusion

In summary, we have formulated a practical and precise nomogram utilizing seven commonly accessible immune-inflammatory-nutritional indicators to predict 28-day mortality in sepsis patients with ARDS. This tool surpasses the SOFA score in performance and provides a more detailed understanding of patient risk. The model has the potential to aid clinicians in the early identification of high-risk patients, thereby enabling the implementation of timely and individualized therapeutic interventions, which could ultimately enhance patient outcomes.

## Data Availability

The raw data supporting the conclusions of this article will be made available by the authors, without undue reservation.
