# Supplementary material for: A risk prediction model based on immune-inflammatory-nutritional indicators for predicting 28-day mortality in sepsis patients with acute respiratory distress syndrome
Source: Front Nutr. 2026 Feb 25;13:1764044. doi: 10.3389/fnut.2026.1764044 (PMC12976859; doi:10.3389/fnut.2026.1764044)
Supplement: Supplementary file 1 [file Data_Sheet_1.zip › Supplementary File 3.docx]

**Supplementary File 3: Nomogram Implementation Details**

**1. Model Coefficients and Intercept**

The logistic regression model for predicting 28‑day mortality is defined as:

where the coefficients are:

| Variable | Coefficient (β) |
| --- | --- |
| Intercept | 0.144 |
| AAPR | –2.203 |
| ALBI | 0.619 |
| NLR | 0.016 |
| PLR | –0.004 |
| PNI | –0.056 |
| SII | 2.38664×10⁻⁵ |
| LAR | 10.585 |

**2. Variable Definitions and Units**

AAPR: albumin (g/L) / alkaline phosphatase (IU/L)

ALBI: albumin-bilirubin grade (1, 2, or 3)

NLR: neutrophil count / lymphocyte count (both in ×10⁹/L)

PLR: platelet count (×10⁹/L) / lymphocyte count (×10⁹/L)

PNI: albumin (g/L) + 5 × lymphocyte count (×10⁹/L)

SII: platelet count × neutrophil count / lymphocyte count (all in ×10⁹/L)

LAR: lactate (mmol/L) / albumin (g/L)

**3. Instructions for Applying the Nomogram to New Data**

1. Ensure all seven variables are collected within the first 24 hours of ICU admission.
2. Calculate each composite indicator as defined above.
3. Use the nomogram (Figure 3) to assign points for each variable based on its value.
4. Sum all points and locate the total on the “Total Points” axis.
5. Project downward to the “Risk of 28-day mortality” axis to obtain the predicted probability.
6. Alternatively, the probability can be computed directly using the logistic formula:
7. P=where is calculated using the coefficients above.

**4. Sample Data and Code**

A sample dataset (sample_data.csv) and R script are provided in the Supplementary File 1 and 2 to illustrate the calculation process and enable validation.
