# Supplementary material for: A risk prediction model based on immune-inflammatory-nutritional indicators for predicting 28-day mortality in sepsis patients with acute respiratory distress syndrome
Source: Front Nutr. 2026 Feb 25;13:1764044. doi: 10.3389/fnut.2026.1764044 (PMC12976859; doi:10.3389/fnut.2026.1764044)
Supplement: Supplementary file 1 [file Data_Sheet_1.zip › Supplementary File 1 - Chinese Version.docx]

#################################################################

#### 欢迎使用“医学列线图平台” 小程序在临床工作中使用列线图！ ####

#### 也欢迎大家关注“医学列线图”公众号 ####

#### 我们经常推送各种列线图教程及优质的列线图研究文章！ ####

#### 欢迎关注，一起做图！ ####

#### 作者：马驰原教授团队、刘玉秀教授团队 ####

#################################################################

#关于这些代码的使用方法，我们近期也会推出视频讲解课程，欢迎大家持续关注“医学列线图”公众号，相关信息和资源会在公众号中发布。

#我们还开发了“医学列线图”微信小程序，可以线上使用小程序收录的列线图。

#有任何问题欢迎联系nomogramhelp@126.com

#################################################################################

#### 以下代码完成一键对数据集进行随机化分为训练集和验证集 ####

#### 并完成组间差异的统计检验 ####

#### 1.连续型变量根据正态性和方差齐性选择t检验或Wilcox秩和检验 ####

#### 2.分类数据（包括二分类和多分类）根据数据情况选择卡方检验或fisher精确检验####

#### 3.生存数据用log rank检验 ####

#################################################################################

#tips:1.所有的文件路径包括文件名尽量不要出现中文字符及空格。可以用下划线_代替空格区分单词。

# 2.报错里是warning没关系，还可以往下运行。如果是error就要停下来查看原因，一味往下运行是解决不了问题的。

################ 0.环境准备及数据输入 ##########################

##0.0 安装需要的R包

#（第一次使用时才需要运行此部分代码。安装成功后不需要重复安装）

install.packages('car')

install.packages('survival')

##0.1 调用R包

#清除R中的所有数据（归零）

rm(list = ls())

library(car)

library(survival)

##0.2 设置数据存放的文件夹目录

#注意文件夹路径的斜杠是双斜杠\\

data_dir <- choose.dir(default = "D:\\data_dir", caption = "选择数据存放的文件夹目录")

##0.3 设置导出文件的文件夹目录

#注意文件夹路径的斜杠是双斜杠\\

output_dir <- choose.dir(default = "D:\\output_dir", caption = "选择导出文件的文件夹目录")

##0.4 选择完整数据集数据文件（模板为logistic_all_dataset.txt或者cox_all_dataset.txt）

#根据logistic和cox分析使用的不同数据，请根据以下要求设置好导入的数据文件格式：

#对于logistic数据：

#数据文件格式要求：注意数据文件要保存为txt版本

#数据格式要求：1. 数据中不要有中文字符；

# 2. 数据中第一行为变量名；

# 3. 数据中第一列为预测结局二分类指标（结局指标必须设置为0/1形式）；

# 4. 数据中从第二列开始为其他变量；

# 5. 请保持训练集和验证集中相同变量的名称一致

# 6. 空值以NA填充；二分类变量以0/1表示；多分类变量进行哑变量处理后以0/1表示；

#模板为生成的仿真数据

#---------------------------------#

#对于cox数据：

#数据文件格式要求：注意数据文件要保存为txt版本

#数据格式要求：1. 数据中不要有中文字符；

# 2. 数据中第一行为变量名；

# 3. 数据中第一列和第二列为预测生存结局的时间和结局事件发生与否的指标（结局指标必须设置为0/1形式）（本套代码中必须以“OS”和"Censor"为结局事件变量名）；

# 4. 数据中从第三列开始为其他变量；

# 5. 请保持训练集和验证集中相同变量的名称一致

# 6. 空值以NA填充；二分类变量以0/1表示；多分类变量进行哑变量处理后以0/1表示；

#模板为生成的仿真数据

#此外，这套代码额外增加第二行类型注释行，用来注释对应每一列变量的类型。

#结局事件为“outcome”；连续型变量为“continuous_variable”；二分类型变量为“binary_variable”；

#此外，若有多分类变量，则第一个多分类变量下的所有哑变量类型均为“polytomous_variable_1”，第二个多分类变量下的所有哑变量类型均为“polytomous_variable_2”，有更多多分类变量则以此类推。

#选择文件路径

all_dataset_path <- choose.files(default = data_dir, caption = "请选数据集数据文件的txt文件。",

multi = TRUE, filters = Filters,

index = nrow(Filters))

#读取数据

all_dataset<- read.csv(all_dataset_path, header = TRUE,sep="\t", stringsAsFactors=FALSE)

#查看数据（一定要运行这一步）

if(TRUE){

variables_label<-all_dataset[1,]

clean_dataset<-apply(all_dataset[-1,],2, as.numeric)

if(length(which(variables_label[1,]=="outcome"))==0){

print("您尚未设置结局变量标签outcome,请参考模板数据进行设置后重新导入数据")

}else if(length(which(variables_label[1,]=="outcome"))>0){

#打印数据类型

if(length(which(variables_label[1,]=="outcome"))==1){

data_type<-"logistic_data_type"

print(paste0("您的结局数据为 二分类 数据，需进行logistic回归分析"))

}else if(length(which(variables_label[1,]=="outcome"))==2){

data_type<-"cox_data_type"

print(paste0("您的结局数据为 生存类型 数据，需进行cox回归分析"))

}else if(length(which(variables_label[1,]=="outcome"))>2){

print("您尚设置结局变量标签outcome超过2个,请参考模板数据进行设置后重新导入数据")

}

#打印总的样本数

print(paste0("您的数据中共包含 ",dim(clean_dataset)[1]," 个样本"))

#打印连续型变量数据情况

if(length(which(variables_label[1,]=="continuous_variable"))==0){

print(paste0("您数据中包含 0 个连续型变量"))

}else if(length(which(variables_label[1,]=="continuous_variable"))>0){

continuous_variable_names<-colnames(variables_label)[which(variables_label[1,]=="continuous_variable")]

print(paste0("您数据中包含 ",length(continuous_variable_names)," 个连续型变量如下："))

for (index_continuous_variable_names in 1:length(continuous_variable_names)) {

print(paste0(" ",

continuous_variable_names[index_continuous_variable_names],

" 其中缺失值 ",

sum(is.na(clean_dataset[,continuous_variable_names[index_continuous_variable_names]])),

" 个 缺失值占比 ",

round((100*((sum(is.na(clean_dataset[,continuous_variable_names[index_continuous_variable_names]])))/(dim(clean_dataset)[1]))),2),

"%"))

}

}

#打印二分类型变量数据情况

if(length(which(variables_label[1,]=="binary_variable"))==0){

print(paste0("您数据中包含 0 个二分类型变量"))

}else if(length(which(variables_label[1,]=="binary_variable"))>0){

binary_variable_names<-colnames(variables_label)[which(variables_label[1,]=="binary_variable")]

print(paste0("您数据中包含 ",length(binary_variable_names)," 个二分类型变量如下："))

for (index_binary_variable_names in 1:length(binary_variable_names)) {

print(paste0(" ",

binary_variable_names[index_binary_variable_names],

" 其中缺失值 ",

sum(is.na(clean_dataset[,binary_variable_names[index_binary_variable_names]])),

" 个 缺失值占比 ",

round((100*((sum(is.na(clean_dataset[,binary_variable_names[index_binary_variable_names]])))/(dim(clean_dataset)[1]))),2),

"%"))

}

}

#打印多分类型变量数据情况

if(length(grep("polytomous_variable", variables_label[1,]))==0){

print(paste0("您数据中包含 0 个多分类型变量"))

}else if(length(grep("polytomous_variable", variables_label[1,]))>0){

poly_names_table<-table(as.character(variables_label[1,grep("polytomous_variable", variables_label[1,])]))

print(paste0("您数据中包含 ",length(poly_names_table)," 个多分类型变量如下："))

for (index_polytomous_variable in 1:length(poly_names_table)) {

print(paste0("其中第 ",index_polytomous_variable,"个多分类变量："))

temp_index_polytomous_variable_label_name<-paste0("polytomous_variable_",index_polytomous_variable)

for(index_polytomous_variable_detail in 1:length(which(variables_label[1,]==temp_index_polytomous_variable_label_name))){

temp_index_polytomous_variable_name<-colnames(variables_label)[which(variables_label[1,]==temp_index_polytomous_variable_label_name)][index_polytomous_variable_detail]

print(paste0(" ",

temp_index_polytomous_variable_name,

" 其中缺失值 ",

sum(is.na(clean_dataset[,temp_index_polytomous_variable_name])),

" 个 缺失值占比 ",

round((100*((sum(is.na(clean_dataset[,temp_index_polytomous_variable_name])))/(dim(clean_dataset)[1]))),2),

"%"))

}

}

}

}

}

################ 1.进行随机化划分训练集及验证集 ##########################

##1.1设置训练集和验证集的随机化比例

training_rate<-3

validation_rate<-1

##1.2进行随机化并进行组间检验

#set.seed(1234)#设置随机化种子

{#设置随机顺序

rand_seq <- 1:dim(clean_dataset)[1]

rand_seq <-sample(rand_seq)

#计算训练集个数

training_count<-ceiling(dim(clean_dataset)[1]*((training_rate)/(training_rate+validation_rate)))

#划分训练集及验证集

temp_trainging_dataset<-clean_dataset[(rand_seq[1:training_count]),]

temp_validation_dataset<-clean_dataset[(rand_seq[(training_count+1):dim(clean_dataset)[1]]),]

##1.3两组间变量比较

#生成空的比较结果汇总表格

printed_results_table<-data.frame(variables="variables",training_dataset="training_dataset",validation_dataset="validation_dataset",p_value="p_value",stringsAsFactors=FALSE)

#比较outcome

if("outcome" %in% names(table(as.character(variables_label)))){

if(data_type=="logistic_data_type"){

variable_names<-colnames(variables_label)[as.character(variables_label)=="outcome"]

temp_training_data_columes<-temp_trainging_dataset[,variable_names]

temp_validation_data_columes<-temp_validation_dataset[,variable_names]

#生成空M

M <- as.table(matrix(0,2,(length(variable_names)+1)))

dimnames(M) <- list(datasets = c("training", "validation"),

classes = c("ref",variable_names))

#计数并赋给M

#整理training_data中的数据

##ref

M["training","ref"]<-sum((temp_training_data_columes==0), na.rm = T)

##other classes

for (index_classes in 2:dim(M)[2]) {

M["training",colnames(M)[index_classes]]<-sum(temp_training_data_columes==1, na.rm = T)

}

#整理validation_data中的数据

##ref

M["validation","ref"]<-sum((temp_validation_data_columes==0), na.rm = T)

##other classes

for (index_classes in 2:dim(M)[2]) {

M["validation",colnames(M)[index_classes]]<-sum(temp_validation_data_columes==1, na.rm = T)

}

#计算结果

col_matrix_ratio_producer<-function(M, round_num){

out_table<-M

for(index_row in 1:nrow(M)){

sum_row<-sum(M[index_row,])

for(index_col in 1:ncol(M)){

ratio<-round(100*M[index_row,index_col]/sum_row, round_num)

out_table[index_row,index_col]<-paste0(M[index_row,index_col], " (",ratio,"%)")

}

}

return(out_table)

}

chisqcp<-chisq.test(M)[3][[1]]

fisherp<- tryCatch(fisher.test(M)[1][[1]],error=function(e){return("A")} )

if(fisherp=="A"){ # 判断当前循环的try语句中的表达式是否运行正确

fisherp<-fisher.test(M,simulate.p.value=TRUE)[1][[1]]

# 此处可以对运行错误的情况进行处理应对

}

col_matrix_observed<-chisq.test(M)$observed

col_matrix_expected<-chisq.test(M)$expected

round_num<-1

col_matrix_ratio<-col_matrix_ratio_producer(M, round_num)

##总的观察数

sum_N<-sum(M)

#评估应该选择的是卡方检验还是fisher检验的结果

if(any(col_matrix_expected<5) | sum_N<40){

finalp<-fisherp

}else{

finalp<-chisqcp

}

#增加结果到输出的表格中

temp_printed_results_table<-data.frame(variables=c("ref",variable_names),training_dataset=col_matrix_ratio["training",],validation_dataset=col_matrix_ratio["validation",],p_value=c(finalp,"NA"),stringsAsFactors=FALSE)

printed_results_table<-rbind(printed_results_table,temp_printed_results_table)

}else if(data_type=="cox_data_type"){

#生成sur计算需要的表格

temp_sur_training_table<-temp_trainging_dataset[,c("OS","Censor","Censor")]

colnames(temp_sur_training_table)[3]<-"grp"

temp_sur_training_table[,3]<-0

temp_sur_validation_table<-temp_validation_dataset[,c("OS","Censor","Censor")]

colnames(temp_sur_validation_table)[3]<-"grp"

temp_sur_validation_table[,3]<-1

temp_sur_table<-rbind(temp_sur_training_table,temp_sur_validation_table)

temp_sur_table<-as.data.frame(temp_sur_table)

#logrank检验

sur_comp<-survdiff(Surv(OS, Censor) ~ grp,

data = temp_sur_table)#默认log rank检验

#增加结果到输出的表格中

temp_printed_results_table<-data.frame(variables="Survival",training_dataset="NA",validation_dataset="NA",p_value=sur_comp$chisq,stringsAsFactors=FALSE)

printed_results_table<-rbind(printed_results_table,temp_printed_results_table)

}

}

#比较连续型变量

if("continuous_variable" %in% names(table(as.character(variables_label)))){

all_continuous_variable_names<-colnames(variables_label)[as.character(variables_label)=="continuous_variable"]

for (index_all_continuous_variable_names in 1:length(all_continuous_variable_names)) {

variable_names<-all_continuous_variable_names[index_all_continuous_variable_names]

print(variable_names)

temp_training_data_columes<-temp_trainging_dataset[,variable_names]

temp_validation_data_columes<-temp_validation_dataset[,variable_names]

#去掉NA

temp_training_data_columes<-temp_training_data_columes[!is.na(temp_training_data_columes)]

temp_validation_data_columes<-temp_validation_data_columes[!is.na(temp_validation_data_columes)]

judge_p<-function(p1,p2,p3,tp,zhp){

mark<-0

if(p1<0.05){

mark<-1

}

if(p2<0.05){

mark<-1

}

if(p3<0.05){

mark<-1

}

if(mark==0){

return(tp)

}else{

return(zhp)

}

}

grp_1_ztp<-shapiro.test(temp_training_data_columes)[2][[1]]

grp_2_ztp<-shapiro.test(temp_validation_data_columes)[2][[1]]

y_leveneT=c(temp_training_data_columes, temp_validation_data_columes)

group_leveneT=as.factor(c(rep(1,length(temp_training_data_columes)), rep(2,length(temp_validation_data_columes))))

fcp<-leveneTest(y_leveneT,group_leveneT)[3][[1]][1]

t_testp<-t.test(temp_training_data_columes,temp_validation_data_columes,paired=F)[3][[1]]

df = data.frame(y_leveneT,group_leveneT)

zhp<-wilcox.test(y_leveneT~group_leveneT, df)[3][[1]]

finalp<-judge_p(p1=grp_1_ztp, p2=grp_2_ztp, p3=fcp, tp=t_testp, zhp=zhp)

#增加结果到输出的表格中

temp_training_label<-paste0(round(mean(temp_training_data_columes),2)," ± ",round(sd(temp_training_data_columes),2))

temp_validation_label<-paste0(round(mean(temp_validation_data_columes),2)," ± ",round(sd(temp_validation_data_columes),2))

temp_printed_results_table<-data.frame(variables=variable_names,training_dataset=temp_training_label,validation_dataset=temp_validation_label,p_value=finalp,stringsAsFactors=FALSE)

printed_results_table<-rbind(printed_results_table,temp_printed_results_table)

}

}

#比较二分类型变量

if("binary_variable" %in% names(table(as.character(variables_label)))){

all_binary_variable_names<-colnames(variables_label)[as.character(variables_label)=="binary_variable"]

for (index_all_binary_variable_names in 1:length(all_binary_variable_names)) {

variable_names<-all_binary_variable_names[index_all_binary_variable_names]

temp_training_data_columes<-temp_trainging_dataset[,variable_names]

temp_validation_data_columes<-temp_validation_dataset[,variable_names]

#生成空M

M <- as.table(matrix(0,2,(length(variable_names)+1)))

dimnames(M) <- list(datasets = c("training", "validation"),

classes = c("ref",variable_names))

#计数并赋给M

#整理training_data中的数据

##ref

M["training","ref"]<-sum((temp_training_data_columes==0), na.rm = T)

##other classes

for (index_classes in 2:dim(M)[2]) {

M["training",colnames(M)[index_classes]]<-sum(temp_training_data_columes==1, na.rm = T)

}

#整理validation_data中的数据

##ref

M["validation","ref"]<-sum((temp_validation_data_columes==0), na.rm = T)

##other classes

for (index_classes in 2:dim(M)[2]) {

M["validation",colnames(M)[index_classes]]<-sum(temp_validation_data_columes==1, na.rm = T)

}

#计算结果

col_matrix_ratio_producer<-function(M, round_num){

out_table<-M

for(index_row in 1:nrow(M)){

sum_row<-sum(M[index_row,])

for(index_col in 1:ncol(M)){

ratio<-round(100*M[index_row,index_col]/sum_row, round_num)

out_table[index_row,index_col]<-paste0(M[index_row,index_col], " (",ratio,"%)")

}

}

return(out_table)

}

chisqcp<-chisq.test(M)[3][[1]]

fisherp<- tryCatch(fisher.test(M)[1][[1]],error=function(e){return("A")} )

if(fisherp=="A"){ # 判断当前循环的try语句中的表达式是否运行正确

fisherp<-fisher.test(M,simulate.p.value=TRUE)[1][[1]]

# 此处可以对运行错误的情况进行处理应对

}

col_matrix_observed<-chisq.test(M)$observed

col_matrix_expected<-chisq.test(M)$expected

round_num<-1

col_matrix_ratio<-col_matrix_ratio_producer(M, round_num)

##总的观察数

sum_N<-sum(M)

#评估应该选择的是卡方检验还是fisher检验的结果

if(any(col_matrix_expected<5) | sum_N<40){

finalp<-fisherp

}else{

finalp<-chisqcp

}

#增加结果到输出的表格中

temp_printed_results_table<-data.frame(variables=c("ref",variable_names),training_dataset=col_matrix_ratio["training",],validation_dataset=col_matrix_ratio["validation",],p_value=c(finalp,"NA"),stringsAsFactors=FALSE)

printed_results_table<-rbind(printed_results_table,temp_printed_results_table)

}

}

#比较多分类型变量

if(length(grep("polytomous_variable", names(table(as.character(variables_label)))))>0){

for(index_polytomous_variable in 1:length(grep("polytomous_variable", names(table(as.character(variables_label)))))){

polytomous_variable_names<-paste0("polytomous_variable_",index_polytomous_variable)

variable_names<-colnames(variables_label)[variables_label[1,]==polytomous_variable_names]

temp_training_data_columes<-temp_trainging_dataset[,variable_names]

temp_validation_data_columes<-temp_validation_dataset[,variable_names]

#生成空M

M <- as.table(matrix(0,2,(length(variable_names)+1)))

dimnames(M) <- list(datasets = c("training", "validation"),

classes = c("ref",variable_names))

#计数并赋给M

#整理training_data中的数据

##ref

M["training","ref"]<-sum(apply(temp_training_data_columes,1,function(vec){all(vec==0)}), na.rm = T)

##other classes

for (index_classes in 2:dim(M)[2]) {

M["training",colnames(M)[index_classes]]<-sum(temp_training_data_columes[,colnames(M)[index_classes]], na.rm = T)

}

#整理validation_data中的数据

##ref

M["validation","ref"]<-sum(apply(temp_validation_data_columes,1,function(vec){all(vec==0)}), na.rm = T)

##other classes

for (index_classes in 2:dim(M)[2]) {

M["validation",colnames(M)[index_classes]]<-sum(temp_validation_data_columes[,colnames(M)[index_classes]], na.rm = T)

}

#计算结果

col_matrix_ratio_producer<-function(M, round_num){

out_table<-M

for(index_row in 1:nrow(M)){

sum_row<-sum(M[index_row,])

for(index_col in 1:ncol(M)){

ratio<-round(100*M[index_row,index_col]/sum_row, round_num)

out_table[index_row,index_col]<-paste0(M[index_row,index_col], " (",ratio,"%)")

}

}

return(out_table)

}

chisqcp<-chisq.test(M)[3][[1]]

fisherp<- tryCatch(fisher.test(M)[1][[1]],error=function(e){return("A")} )

if(fisherp=="A"){ # 判断当前循环的try语句中的表达式是否运行正确

fisherp<-fisher.test(M,simulate.p.value=TRUE)[1][[1]]

# 此处可以对运行错误的情况进行处理应对

}

col_matrix_observed<-chisq.test(M)$observed

col_matrix_expected<-chisq.test(M)$expected

round_num<-1

col_matrix_ratio<-col_matrix_ratio_producer(M, round_num)

##总的观察数

sum_N<-sum(M)

#评估应该选择的是卡方检验还是fisher检验的结果

if(any(col_matrix_expected<5) | sum_N<40){

finalp<-fisherp

}else{

finalp<-chisqcp

}

#增加结果到输出的表格中

temp_printed_results_table<-data.frame(variables=c("ref",variable_names),training_dataset=col_matrix_ratio["training",],validation_dataset=col_matrix_ratio["validation",],p_value=c(finalp,rep("NA",(dim(col_matrix_ratio)[2]-1))),stringsAsFactors=FALSE)

printed_results_table<-rbind(printed_results_table,temp_printed_results_table)

}

}

#去掉空的第一行

printed_results_table<-printed_results_table[-1,]

#计算小于0.05的p值个数

all_p_value<-as.numeric(printed_results_table$p_value)

all_p_value<-sum(all_p_value<=0.05,na.rm = TRUE)}

######

##1.3打印报告

{print("该次随机化分组的训练集及验证集间比较如下：")

print(paste0("其中，有 ",all_p_value," 个变量存在组间差异（p值<=0.05）"))

print(printed_results_table)}

##1.4导出训练集及验证集数据及数据集间比较结果

#如要导出训练集、验证集数据及数据集间比较结果，请运行下面一行代码。

#如对随机化结果不满意，请再次运行 1.2进行随机化 步骤

write.table(temp_trainging_dataset,file = paste0(output_dir, "\\", "randomized_trainging_dataset.txt"), row.names = FALSE,sep = "\t")

write.table(temp_validation_dataset,file = paste0(output_dir, "\\", "randomized_validation_dataset.txt"), row.names = FALSE,sep = "\t")

write.table(printed_results_table,file = paste0(output_dir, "\\", "randomized_comparison_result.txt"), row.names = FALSE,sep = "\t")
