# Supplementary material for: A risk prediction model based on immune-inflammatory-nutritional indicators for predicting 28-day mortality in sepsis patients with acute respiratory distress syndrome
Source: Front Nutr. 2026 Feb 25;13:1764044. doi: 10.3389/fnut.2026.1764044 (PMC12976859; doi:10.3389/fnut.2026.1764044)
Supplement: Supplementary file 1 [file Data_Sheet_1.zip › Supplementary File 1 - English Version.docx]

################################################################

#### Welcome to the "Medical Nomogram Platform" WeChat Mini Program for using nomograms in clinical practice! ####

#### Also, welcome to follow the "Medical Nomogram" WeChat Official Account ####

#### We regularly share various nomogram tutorials and high-quality nomogram research articles! ####

#### Welcome to follow us and create nomograms together! ####

#### Authors: Team of Professor Chiyuan Ma, Team of Professor Yuxiu Liu ####

################################################################

# For instructions on using this code, we will soon release video tutorial courses.

# Welcome to continue following the "Medical Nomogram" WeChat Official Account for updates and resources.

# We have also developed the "Medical Nomogram" WeChat Mini Program for online use of included nomograms.

# For any questions, please contact nomogramhelp@126.com

#################################################################################

#### The following code performs one-click randomization of a dataset into training and validation sets ####

#### and conducts statistical comparisons between groups ####

#### 1. For continuous variables, t-test or Wilcoxon rank-sum test is selected based on normality and homogeneity of variance ####

#### 2. For categorical data (including binary and multi-category), chi-square test or Fisher's exact test is selected based on data conditions ####

#### 3. For survival data, log-rank test is used ####

#################################################################################

# Tips: 1. Avoid using Chinese characters and spaces in file paths and names. Use underscores _ instead of spaces to separate words.

# 2. Warnings in error messages are acceptable; the code can continue running. If an error occurs, stop and investigate the cause. Continuing blindly will not solve the problem.

################ 0. Environment Preparation and Data Input ##########################

## 0.0 Install required R packages

# (Run this section only for the first use. No need to repeat after successful installation)

install.packages('car')

install.packages('survival')

## 0.1 Load R packages

# Clear all data in R (reset)

rm(list = ls())

library(car)

library(survival)

## 0.2 Set the directory for data storage

# Note: Use double backslashes \\ for folder paths

data_dir <- choose.dir(default = "D:\\data_dir", caption = "Select the folder directory for data storage")

## 0.3 Set the directory for exporting files

# Note: Use double backslashes \\ for folder paths

output_dir <- choose.dir(default = "D:\\output_dir", caption = "Select the folder directory for exporting files")

## 0.4 Select the complete dataset file (templates: logistic_all_dataset.txt or cox_all_dataset.txt)

# For logistic and Cox analysis data, please set the imported data file format according to the following requirements:

# For logistic data:

# Data file format requirements: Save the data file as a txt version.

# Data format requirements: 1. Avoid Chinese characters in the data;

# 2. The first row of the data should contain variable names;

# 3. The first column should be the binary outcome indicator (must be in 0/1 format);

# 4. Other variables should start from the second column;

# 5. Keep variable names consistent between training and validation sets;

# 6. Fill missing values with NA; represent binary variables as 0/1; represent multi-category variables as dummy variables (0/1).

# Template: Generated simulated data.

# For Cox data:

# Data file format requirements: Save the data file as a txt version.

# Data format requirements: 1. Avoid Chinese characters in the data;

# 2. The first row of the data should contain variable names;

# 3. The first and second columns should be the time-to-event and event indicator (must be in 0/1 format) (variable names must be "OS" and "Censor" in this code);

# 4. Other variables should start from the third column;

# 5. Keep variable names consistent between training and validation sets;

# 6. Fill missing values with NA; represent binary variables as 0/1; represent multi-category variables as dummy variables (0/1).

# Template: Generated simulated data.

# Additionally, this code includes an extra second row for type annotations to specify the type of each variable.

# Outcome event: "outcome"; continuous variable: "continuous_variable"; binary variable: "binary_variable";

# For multi-category variables, all dummy variables under the first multi-category variable are labeled as "polytomous_variable_1",

# the second as "polytomous_variable_2", and so on for more multi-category variables.

# Select file path

all_dataset_path <- choose.files(default = data_dir, caption = "Select the txt file for the dataset.",

multi = TRUE, filters = Filters,

index = nrow(Filters))

# Read data

all_dataset <- read.csv(all_dataset_path, header = TRUE, sep = "\t", stringsAsFactors = FALSE)

# View data (this step must be run)

if (TRUE) {

variables_label <- all_dataset[1, ]

clean_dataset <- apply(all_dataset[-1, ], 2, as.numeric)

if (length(which(variables_label[1, ] == "outcome")) == 0) {

print("You have not set the outcome variable label 'outcome'. Please refer to the template data and re-import the data.")

} else if (length(which(variables_label[1, ] == "outcome")) > 0) {

# Print data type

if (length(which(variables_label[1, ] == "outcome")) == 1) {

data_type <- "logistic_data_type"

print(paste0("Your outcome data is binary and requires logistic regression analysis."))

} else if (length(which(variables_label[1, ] == "outcome")) == 2) {

data_type <- "cox_data_type"

print(paste0("Your outcome data is survival-type and requires Cox regression analysis."))

} else if (length(which(variables_label[1, ] == "outcome")) > 2) {

print("You have set more than 2 outcome variable labels. Please refer to the template data and re-import the data.")

}

# Print total sample size

print(paste0("Your data contains ", dim(clean_dataset)[1], " samples."))

# Print continuous variable data

if (length(which(variables_label[1, ] == "continuous_variable")) == 0) {

print(paste0("Your data contains 0 continuous variables."))

} else if (length(which(variables_label[1, ] == "continuous_variable")) > 0) {

continuous_variable_names <- colnames(variables_label)[which(variables_label[1, ] == "continuous_variable")]

print(paste0("Your data contains ", length(continuous_variable_names), " continuous variables as follows:"))

for (index_continuous_variable_names in 1:length(continuous_variable_names)) {

print(paste0(" ",

continuous_variable_names[index_continuous_variable_names],

" Missing values: ",

sum(is.na(clean_dataset[, continuous_variable_names[index_continuous_variable_names]])),

" Proportion of missing values: ",

round((100 * ((sum(is.na(clean_dataset[, continuous_variable_names[index_continuous_variable_names]]))) / (dim(clean_dataset)[1]))), 2),

"%"))

}

}

# Print binary variable data

if (length(which(variables_label[1, ] == "binary_variable")) == 0) {

print(paste0("Your data contains 0 binary variables."))

} else if (length(which(variables_label[1, ] == "binary_variable")) > 0) {

binary_variable_names <- colnames(variables_label)[which(variables_label[1, ] == "binary_variable")]

print(paste0("Your data contains ", length(binary_variable_names), " binary variables as follows:"))

for (index_binary_variable_names in 1:length(binary_variable_names)) {

print(paste0(" ",

binary_variable_names[index_binary_variable_names],

" Missing values: ",

sum(is.na(clean_dataset[, binary_variable_names[index_binary_variable_names]])),

" Proportion of missing values: ",

round((100 * ((sum(is.na(clean_dataset[, binary_variable_names[index_binary_variable_names]]))) / (dim(clean_dataset)[1]))), 2),

"%"))

}

}

# Print multi-category variable data

if (length(grep("polytomous_variable", variables_label[1, ])) == 0) {

print(paste0("Your data contains 0 multi-category variables."))

} else if (length(grep("polytomous_variable", variables_label[1, ])) > 0) {

poly_names_table <- table(as.character(variables_label[1, grep("polytomous_variable", variables_label[1, ])]))

print(paste0("Your data contains ", length(poly_names_table), " multi-category variables as follows:"))

for (index_polytomous_variable in 1:length(poly_names_table)) {

print(paste0(" For the ", index_polytomous_variable, "th multi-category variable:"))

temp_index_polytomous_variable_label_name <- paste0("polytomous_variable_", index_polytomous_variable)

for (index_polytomous_variable_detail in 1:length(which(variables_label[1, ] == temp_index_polytomous_variable_label_name))) {

temp_index_polytomous_variable_name <- colnames(variables_label)[which(variables_label[1, ] == temp_index_polytomous_variable_label_name)][index_polytomous_variable_detail]

print(paste0(" ",

temp_index_polytomous_variable_name,

" Missing values: ",

sum(is.na(clean_dataset[, temp_index_polytomous_variable_name])),

" Proportion of missing values: ",

round((100 * ((sum(is.na(clean_dataset[, temp_index_polytomous_variable_name]))) / (dim(clean_dataset)[1]))), 2),

"%"))

}

}

}

}

}

################ 1. Randomization into Training and Validation Sets ##########################

## 1.1 Set randomization ratio for training and validation sets

training_rate <- 3

validation_rate <- 1

## 1.2 Perform randomization and conduct between-group tests

# set.seed(1234) # Set random seed (optional)

{

# Set random order

rand_seq <- 1:dim(clean_dataset)[1]

rand_seq <- sample(rand_seq)

# Calculate number of training samples

training_count <- ceiling(dim(clean_dataset)[1] * ((training_rate) / (training_rate + validation_rate)))

# Divide into training and validation sets

temp_training_dataset <- clean_dataset[(rand_seq[1:training_count]), ]

temp_validation_dataset <- clean_dataset[(rand_seq[(training_count + 1):dim(clean_dataset)[1]]), ]

## 1.3 Compare variables between groups

# Generate empty summary table for comparison results

printed_results_table <- data.frame(variables = "variables", training_dataset = "training_dataset", validation_dataset = "validation_dataset", p_value = "p_value", stringsAsFactors = FALSE)

# Compare outcome

if ("outcome" %in% names(table(as.character(variables_label)))) {

if (data_type == "logistic_data_type") {

variable_names <- colnames(variables_label)[as.character(variables_label) == "outcome"]

temp_training_data_columes <- temp_training_dataset[, variable_names]

temp_validation_data_columes <- temp_validation_dataset[, variable_names]

# Generate empty M

M <- as.table(matrix(0, 2, (length(variable_names) + 1)))

dimnames(M) <- list(datasets = c("training", "validation"),

classes = c("ref", variable_names))

# Count and assign to M

# For training data

M["training", "ref"] <- sum((temp_training_data_columes == 0), na.rm = T)

for (index_classes in 2:dim(M)[2]) {

M["training", colnames(M)[index_classes]] <- sum(temp_training_data_columes == 1, na.rm = T)

}

# For validation data

M["validation", "ref"] <- sum((temp_validation_data_columes == 0), na.rm = T)

for (index_classes in 2:dim(M)[2]) {

M["validation", colnames(M)[index_classes]] <- sum(temp_validation_data_columes == 1, na.rm = T)

}

# Calculate results

col_matrix_ratio_producer <- function(M, round_num) {

out_table <- M

for (index_row in 1:nrow(M)) {

sum_row <- sum(M[index_row, ])

for (index_col in 1:ncol(M)) {

ratio <- round(100 * M[index_row, index_col] / sum_row, round_num)

out_table[index_row, index_col] <- paste0(M[index_row, index_col], " (", ratio, "%)")

}

}

return(out_table)

}

chisqcp <- chisq.test(M)[3][[1]]

fisherp <- tryCatch(fisher.test(M)[1][[1]], error = function(e) { return("A") })

if (fisherp == "A") {

fisherp <- fisher.test(M, simulate.p.value = TRUE)[1][[1]]

}

col_matrix_observed <- chisq.test(M)$observed

col_matrix_expected <- chisq.test(M)$expected

round_num <- 1

col_matrix_ratio <- col_matrix_ratio_producer(M, round_num)

# Total observations

sum_N <- sum(M)

# Decide between chi-square and Fisher's test

if (any(col_matrix_expected < 5) | sum_N < 40) {

finalp <- fisherp

} else {

finalp <- chisqcp

}

# Add results to output table

temp_printed_results_table <- data.frame(variables = c("ref", variable_names), training_dataset = col_matrix_ratio["training", ], validation_dataset = col_matrix_ratio["validation", ], p_value = c(finalp, "NA"), stringsAsFactors = FALSE)

printed_results_table <- rbind(printed_results_table, temp_printed_results_table)

} else if (data_type == "cox_data_type") {

# Generate table for survival calculation

temp_sur_training_table <- temp_training_dataset[, c("OS", "Censor", "Censor")]

colnames(temp_sur_training_table)[3] <- "grp"

temp_sur_training_table[, 3] <- 0

temp_sur_validation_table <- temp_validation_dataset[, c("OS", "Censor", "Censor")]

colnames(temp_sur_validation_table)[3] <- "grp"

temp_sur_validation_table[, 3] <- 1

temp_sur_table <- rbind(temp_sur_training_table, temp_sur_validation_table)

temp_sur_table <- as.data.frame(temp_sur_table)

# Log-rank test

sur_comp <- survdiff(Surv(OS, Censor) ~ grp, data = temp_sur_table) # Default log-rank test

# Add results to output table

temp_printed_results_table <- data.frame(variables = "Survival", training_dataset = "NA", validation_dataset = "NA", p_value = sur_comp$chisq, stringsAsFactors = FALSE)

printed_results_table <- rbind(printed_results_table, temp_printed_results_table)

}

}

# Compare continuous variables

if ("continuous_variable" %in% names(table(as.character(variables_label)))) {

all_continuous_variable_names <- colnames(variables_label)[as.character(variables_label) == "continuous_variable"]

for (index_all_continuous_variable_names in 1:length(all_continuous_variable_names)) {

variable_names <- all_continuous_variable_names[index_all_continuous_variable_names]

print(variable_names)

temp_training_data_columes <- temp_training_dataset[, variable_names]

temp_validation_data_columes <- temp_validation_dataset[, variable_names]

# Remove NA

temp_training_data_columes <- temp_training_data_columes[!is.na(temp_training_data_columes)]

temp_validation_data_columes <- temp_validation_data_columes[!is.na(temp_validation_data_columes)]

judge_p <- function(p1, p2, p3, tp, zhp) {

mark <- 0

if (p1 < 0.05) {

mark <- 1

}

if (p2 < 0.05) {

mark <- 1

}

if (p3 < 0.05) {

mark <- 1

}

if (mark == 0) {

return(tp)

} else {

return(zhp)

}

}

grp_1_ztp <- shapiro.test(temp_training_data_columes)[2][[1]]

grp_2_ztp <- shapiro.test(temp_validation_data_columes)[2][[1]]

y_leveneT <- c(temp_training_data_columes, temp_validation_data_columes)

group_leveneT <- as.factor(c(rep(1, length(temp_training_data_columes)), rep(2, length(temp_validation_data_columes))))

fcp <- leveneTest(y_leveneT, group_leveneT)[3][[1]][1]

t_testp <- t.test(temp_training_data_columes, temp_validation_data_columes, paired = F)[3][[1]]

df <- data.frame(y_leveneT, group_leveneT)

zhp <- wilcox.test(y_leveneT ~ group_leveneT, df)[3][[1]]

finalp <- judge_p(p1 = grp_1_ztp, p2 = grp_2_ztp, p3 = fcp, tp = t_testp, zhp = zhp)

# Add results to output table

temp_training_label <- paste0(round(mean(temp_training_data_columes), 2), " ± ", round(sd(temp_training_data_columes), 2))

temp_validation_label <- paste0(round(mean(temp_validation_data_columes), 2), " ± ", round(sd(temp_validation_data_columes), 2))

temp_printed_results_table <- data.frame(variables = variable_names, training_dataset = temp_training_label, validation_dataset = temp_validation_label, p_value = finalp, stringsAsFactors = FALSE)

printed_results_table <- rbind(printed_results_table, temp_printed_results_table)

}

}

# Compare binary variables

if ("binary_variable" %in% names(table(as.character(variables_label)))) {

all_binary_variable_names <- colnames(variables_label)[as.character(variables_label) == "binary_variable"]

for (index_all_binary_variable_names in 1:length(all_binary_variable_names)) {

variable_names <- all_binary_variable_names[index_all_binary_variable_names]

temp_training_data_columes <- temp_training_dataset[, variable_names]

temp_validation_data_columes <- temp_validation_dataset[, variable_names]

# Generate empty M

M <- as.table(matrix(0, 2, (length(variable_names) + 1)))

dimnames(M) <- list(datasets = c("training", "validation"),

classes = c("ref", variable_names))

# Count and assign to M

# For training data

M["training", "ref"] <- sum((temp_training_data_columes == 0), na.rm = T)

for (index_classes in 2:dim(M)[2]) {

M["training", colnames(M)[index_classes]] <- sum(temp_training_data_columes == 1, na.rm = T)

}

# For validation data

M["validation", "ref"] <- sum((temp_validation_data_columes == 0), na.rm = T)

for (index_classes in 2:dim(M)[2]) {

M["validation", colnames(M)[index_classes]] <- sum(temp_validation_data_columes == 1, na.rm = T)

}

# Calculate results

col_matrix_ratio_producer <- function(M, round_num) {

out_table <- M

for (index_row in 1:nrow(M)) {

sum_row <- sum(M[index_row, ])

for (index_col in 1:ncol(M)) {

ratio <- round(100 * M[index_row, index_col] / sum_row, round_num)

out_table[index_row, index_col] <- paste0(M[index_row, index_col], " (", ratio, "%)")

}

}

return(out_table)

}

chisqcp <- chisq.test(M)[3][[1]]

fisherp <- tryCatch(fisher.test(M)[1][[1]], error = function(e) { return("A") })

if (fisherp == "A") {

fisherp <- fisher.test(M, simulate.p.value = TRUE)[1][[1]]

}

col_matrix_observed <- chisq.test(M)$observed

col_matrix_expected <- chisq.test(M)$expected

round_num <- 1

col_matrix_ratio <- col_matrix_ratio_producer(M, round_num)

# Total observations

sum_N <- sum(M)

# Decide between chi-square and Fisher's test

if (any(col_matrix_expected < 5) | sum_N < 40) {

finalp <- fisherp

} else {

finalp <- chisqcp

}

# Add results to output table

temp_printed_results_table <- data.frame(variables = c("ref", variable_names), training_dataset = col_matrix_ratio["training", ], validation_dataset = col_matrix_ratio["validation", ], p_value = c(finalp, "NA"), stringsAsFactors = FALSE)

printed_results_table <- rbind(printed_results_table, temp_printed_results_table)

}

}

# Compare multi-category variables

if (length(grep("polytomous_variable", names(table(as.character(variables_label))))) > 0) {

for (index_polytomous_variable in 1:length(grep("polytomous_variable", names(table(as.character(variables_label)))))) {

polytomous_variable_names <- paste0("polytomous_variable_", index_polytomous_variable)

variable_names <- colnames(variables_label)[variables_label[1, ] == polytomous_variable_names]

temp_training_data_columes <- temp_training_dataset[, variable_names]

temp_validation_data_columes <- temp_validation_dataset[, variable_names]

# Generate empty M

M <- as.table(matrix(0, 2, (length(variable_names) + 1)))

dimnames(M) <- list(datasets = c("training", "validation"),

classes = c("ref", variable_names))

# Count and assign to M

# For training data

M["training", "ref"] <- sum(apply(temp_training_data_columes, 1, function(vec) { all(vec == 0) }), na.rm = T)

for (index_classes in 2:dim(M)[2]) {

M["training", colnames(M)[index_classes]] <- sum(temp_training_data_columes[, colnames(M)[index_classes]], na.rm = T)

}

# For validation data

M["validation", "ref"] <- sum(apply(temp_validation_data_columes, 1, function(vec) { all(vec == 0) }), na.rm = T)

for (index_classes in 2:dim(M)[2]) {

M["validation", colnames(M)[index_classes]] <- sum(temp_validation_data_columes[, colnames(M)[index_classes]], na.rm = T)

}

# Calculate results

col_matrix_ratio_producer <- function(M, round_num) {

out_table <- M

for (index_row in 1:nrow(M)) {

sum_row <- sum(M[index_row, ])

for (index_col in 1:ncol(M)) {

ratio <- round(100 * M[index_row, index_col] / sum_row, round_num)

out_table[index_row, index_col] <- paste0(M[index_row, index_col], " (", ratio, "%)")

}

}

return(out_table)

}

chisqcp <- chisq.test(M)[3][[1]]

fisherp <- tryCatch(fisher.test(M)[1][[1]], error = function(e) { return("A") })

if (fisherp == "A") {

fisherp <- fisher.test(M, simulate.p.value = TRUE)[1][[1]]

}

col_matrix_observed <- chisq.test(M)$observed

col_matrix_expected <- chisq.test(M)$expected

round_num <- 1

col_matrix_ratio <- col_matrix_ratio_producer(M, round_num)

# Total observations

sum_N <- sum(M)

# Decide between chi-square and Fisher's test

if (any(col_matrix_expected < 5) | sum_N < 40) {

finalp <- fisherp

} else {

finalp <- chisqcp

}

# Add results to output table

temp_printed_results_table <- data.frame(variables = c("ref", variable_names), training_dataset = col_matrix_ratio["training", ], validation_dataset = col_matrix_ratio["validation", ], p_value = c(finalp, rep("NA", (dim(col_matrix_ratio)[2] - 1))), stringsAsFactors = FALSE)

printed_results_table <- rbind(printed_results_table, temp_printed_results_table)

}

}

# Remove the empty first row

printed_results_table <- printed_results_table[-1, ]

# Count p-values less than or equal to 0.05

all_p_value <- as.numeric(printed_results_table$p_value)

all_p_value <- sum(all_p_value <= 0.05, na.rm = TRUE)

}

######

## 1.3 Print report

{

print("Comparison between training and validation sets from this randomization is as follows:")

print(paste0("Among them, ", all_p_value, " variables show between-group differences (p-value <= 0.05)."))

print(printed_results_table)

}

## 1.4 Export training and validation datasets and comparison results

# To export training, validation datasets, and comparison results, run the following line.

# If unsatisfied with the randomization result, run step 1.2 again for re-randomization.

write.table(temp_training_dataset, file = paste0(output_dir, "\\", "randomized_training_dataset.txt"), row.names = FALSE, sep = "\t")

write.table(temp_validation_dataset, file = paste0(output_dir, "\\", "randomized_validation_dataset.txt"), row.names = FALSE, sep = "\t")

write.table(printed_results_table, file = paste0(output_dir, "\\", "randomized_comparison_result.txt"), row.names = FALSE, sep = "\t")
