# Supplementary material for: A risk prediction model based on immune-inflammatory-nutritional indicators for predicting 28-day mortality in sepsis patients with acute respiratory distress syndrome
Source: Front Nutr. 2026 Feb 25;13:1764044. doi: 10.3389/fnut.2026.1764044 (PMC12976859; doi:10.3389/fnut.2026.1764044)
Supplement: Supplementary file 1 [file Data_Sheet_1.zip › Supplementary File 2- Chinese Version.docx]

#################################################################

#### 欢迎使用“医学列线图平台” 小程序在临床工作中使用列线图！ ####

#### 也欢迎大家关注“医学列线图”公众号 ####

#### 我们经常推送各种列线图教程及优质的列线图研究文章！ ####

#### 欢迎关注，一起做图！ ####

#### 作者：马驰原教授团队 ####

#################################################################

#关于这些代码的使用方法，我们近期也会推出视频讲解课程，欢迎大家持续关注“医学列线图”公众号，相关信息和资源会在公众号中发布。

#我们还开发了“医学列线图”微信小程序，可以线上使用小程序收录的列线图。

#有任何问题欢迎联系nomogramhelp@126.com

#################################################################

#### 以下是预测结局为二分类变量的列线图分析代码 ####

#### 包括以下几个主要的步骤 ####

#### 1.训练集与验证集数据之间的比较 ####

#### 2.训练集部内部阳性/阴性结局人群之间的比较 ####

#### 3.在训练集中进行单因素和多因素Logistic回归分析 ####

#### 4.基于多因素Logistic回归分析结果构建列线图 ####

#### 5.评估模型（ROC及校准图） ####

#################################################################

#tips:1.所有的文件路径包括文件名尽量不要出现中文字符及空格。可以用下划线_代替空格区分单词。

# 2.报错里是warning没关系，还可以往下运行。如果是error就要停下来查看原因，一味往下运行是解决不了问题的。

################ 0.环境准备及数据输入 ##########################

##0.0 安装需要的R包

#（第一次使用时才需要运行此部分代码。安装成功后不需要重复安装）

install.packages('car')

install.packages('rms')

install.packages('pROC')

#DecisionCurve包是绘制决策曲线的包，不好安装，可以尝试用以下几种方法进行安装

#1

install.packages('DecisionCurve')

#2

BiocManager::install("DecisionCurve",ask = F,update = F)

#3

library(devtools)

install_github("mdbrown/DecisionCurve")

#4 该方法安装的是1.3的版本

data_dir <- choose.dir(default = "D:\\data_dir", caption = "选择DecisionCurve_1.3.tar.gz存放的文件夹目录")

install.packages(paste0(data_dir,"\\DecisionCurve_1.3.tar.gz"),

repos = NULL,

type = "source")

##0.1 调用R包

#清除R中的所有数据（归零）

rm(list = ls())

library(car)

library(rms)

library(pROC)

library(rmda)

##0.2 设置数据存放的文件夹目录

#注意文件夹路径的斜杠是双斜杠\\

data_dir <- choose.dir(default = "D:\\data_dir", caption = "选择数据存放的文件夹目录")

##0.3 设置导出图片的文件夹目录

#注意文件夹路径的斜杠是双斜杠\\

output_dir <- choose.dir(default = "D:\\output_dir", caption = "选择导出图片的文件夹目录")

##0.4 选择训练集数据文件（模板为logistic_training_dataset.txt）

#数据文件格式要求：注意数据文件要保存为txt版本

#数据格式要求：1. 数据中不要有中文字符；

# 2. 数据中第一行为变量名；

# 3. 数据中第一列为预测结局二分类指标（结局指标必须设置为0/1形式）；

# 4. 数据中从第二列开始为其他变量；

# 5. 请保持训练集和验证集中相同变量的名称一致

# 6. 空值以NA填充；二分类变量以0/1表示；多分类变量进行哑变量处理后以0/1表示；

#模板为生成的仿真数据

#选择文件路径

training_dataset_path <- choose.files(default = data_dir, caption = "请选训练集数据文件的txt文件。",

multi = TRUE, filters = Filters,

index = nrow(Filters))

#读取数据

training_dataset<- read.csv(training_dataset_path, header = TRUE,sep="\t", stringsAsFactors=FALSE)

#查看数据

print(paste0("该训练集有 ",dim(training_dataset)[1]," 个样本； ",dim(training_dataset)[2]," 个变量"))

##0.5 选择验证集数据文件（模板为logistic_validation_dataset.txt）

#数据文件格式要求：注意数据文件要保存为txt版本

#数据格式要求：1. 数据中不要有中文字符；

# 2. 数据中第一行为变量名；

# 3. 数据中第一列为预测结局二分类指标（结局指标必须设置为0/1形式）；

# 4. 数据中从第二列开始为其他变量；

# 5. 请保持训练集和验证集中相同变量的名称一致

# 6. 空值以NA填充；二分类变量以0/1表示；多分类变量进行哑变量处理后以0/1表示；

#模板为生成的仿真数据

#选择文件路径

validation_dataset_path <- choose.files(default = data_dir, caption = "请选训练集数据文件的txt文件。",

multi = TRUE, filters = Filters,

index = nrow(Filters))

#读取数据

validation_dataset<- read.csv(validation_dataset_path, header = TRUE,sep="\t", stringsAsFactors=FALSE)

#查看数据

print(paste0("该验证集有 ",dim(validation_dataset)[1]," 个样本； ",dim(validation_dataset)[2]," 个变量"))

################ 1.训练集与验证集数据之间的比较 ##########################

com_type="btw_datasets"

var_name="Gender"

#定义运算函数（这个定义过程请直接运行，不用修改）

comparison_nomogram<-function(com_type,training_dataset,validation_dataset,var_name,var_type){

#com_type:btw_datasets(训练集和验证集之间比较);btw_grp(训练集内部阴性/阳性结局组之间比较,仅适用二分类变量结局)

#training_dataset:训练集数据

#validation_dataset:验证集数据

#var_name:比较的变量名称

#vartype:比较的变量类型：continue_type(连续型变量)；bi_type(二分类型变量)

#提取数据

if(com_type=="btw_datasets"){

data_1=training_dataset[,var_name]

data_2=validation_dataset[,var_name]

}else if(com_type=="btw_grp"){

data_1=training_dataset[(training_dataset[,1]==0),var_name]

data_2=training_dataset[(training_dataset[,1]==1),var_name]

}

#计算数据

if(var_type=="continue_type"){

judge_p<-function(p1,p2,p3,tp,zhp){

mark<-0

if(p1<0.05){

mark<-1

}

if(p2<0.05){

mark<-1

}

if(p3<0.05){

mark<-1

}

if(mark==0){

return(tp)

}else{

return(zhp)

}

}

grp_1_ztp<-shapiro.test(data_1)[2][[1]]

grp_2_ztp<-shapiro.test(data_2)[2][[1]]

y_leveneT=c(data_1, data_2)

group_leveneT=as.factor(c(rep(1,length(data_1)), rep(2,length(data_2))))

fcp<-leveneTest(y_leveneT,group_leveneT)[3][[1]][1]

t_testp<-t.test(data_1,data_2,paired=F)[3][[1]]

df = data.frame(y_leveneT,group_leveneT)

zhp<-wilcox.test(y_leveneT~group_leveneT, df)[3][[1]]

finalp<-judge_p(p1=grp_1_ztp, p2=grp_2_ztp, p3=fcp, tp=t_testp, zhp=zhp)

}else if(var_type=="bi_type"){

#相关函数

col_matrix_producer<-function(grp_0_dingxing_data,grp_1_dingxing_data){

vars<-unique(c(names(table(grp_0_dingxing_data)),names(table(grp_1_dingxing_data))))

col_matrix<-matrix(data = 0, nrow = length(vars), ncol = 2, byrow = FALSE,dimnames = NULL)

colnames(col_matrix)<-c("grp_0","grp_1")

rownames(col_matrix)<-vars

for(index_var in 1:length(vars)){

if(!is.na(table(grp_0_dingxing_data)[vars[index_var]][[1]])){

col_matrix[vars[index_var],"grp_0"]<-table(grp_0_dingxing_data)[vars[index_var]][[1]]

}

}

for(index_var in 1:length(vars)){

if(!is.na(table(grp_1_dingxing_data)[vars[index_var]][[1]])){

col_matrix[vars[index_var],"grp_1"]<-table(grp_1_dingxing_data)[vars[index_var]][[1]]

}

}

return(col_matrix)

}

col_matrix_ratio_producer<-function(col_matrix, round_num){

out_table<-col_matrix

for(index_col in 1:ncol(col_matrix)){

sum_col<-sum(col_matrix[,index_col])

for(index_row in 1:nrow(col_matrix)){

ratio<-round(100*col_matrix[index_row,index_col]/sum_col, round_num)

out_table[index_row,index_col]<-paste0(col_matrix[index_row,index_col], " (",ratio,"%)")

}

}

return(out_table)

}

##去掉可能存在的空值

b0 = which(data_1=="NA")

if(length(b0)){

data_1<-data_1[-b0]

}

b1 = which(data_2=="NA")

if(length(b1)){

data_2<-data_2[-b1]

}

#生成四格表

col_matrix<-col_matrix_producer(data_1,data_2)

#计算结果

chisqcp<-chisq.test(col_matrix)[3][[1]]

fisherp<- tryCatch(fisher.test(col_matrix)[1][[1]],error=function(e){return("A")} )

if(fisherp=="A"){ # 判断当前循环的try语句中的表达式是否运行正确

fisherp<-fisher.test(col_matrix,simulate.p.value=TRUE)[1][[1]]

# 此处可以对运行错误的情况进行处理应对

}

#fisherp<-fisher.test(col_matrix)[1][[1]]

col_matrix_observed<-chisq.test(col_matrix)$observed

col_matrix_expected<-chisq.test(col_matrix)$expected

round_num<-1

col_matrix_ratio<-col_matrix_ratio_producer(col_matrix, round_num)

##总的观察数

sum_N<-sum(col_matrix)

#评估应该选择的是卡方检验还是fisher检验的结果

if(any(col_matrix_expected<5) | sum_N<40){

finalp<-fisherp

}else{

finalp<-chisqcp

}

}

return(paste0("变量",var_name,"对应的p值为：",finalp))

}

#由于不同的数据类型要应用不同的统计学方法比较，因此这里需要你一个个变量进行比较，同时每一次比较都要设置这个变量的类别。

#设置是训练集和验证集之间比较 btw_datasets 还是 训练集内部阴性/阳性结局组之间比较(仅适用二分类变量结局) btw_grp

com_type="btw_datasets"

#设置要比较的变量名

var_name = "age"

#设置该变量名的类别为：continue_type(连续型变量)；bi_type(二分类型变量)

var_type = "continue_type"

#计算

#com_type:btw_datasets(训练集和验证集之间比较);btw_grp(训练集内部阴性/阳性结局组之间比较,仅适用二分类变量结局)

#training_dataset:训练集数据

#validation_dataset:验证集数据

#var_name:比较的变量名称

#vartype:比较的变量类型：continue_type(连续型变量)；bi_type(二分类型变量)

comparison_nomogram(com_type=com_type,

training_dataset=training_dataset,

validation_dataset=validation_dataset,

var_name=var_name,

var_type=var_type)

#### 2.训练集部内部阳性/阴性结局人群之间的比较

#设置是训练集和验证集之间比较 btw_datasets 还是 训练集内部阴性/阳性结局组之间比较(仅适用二分类变量结局) btw_grp

com_type="btw_grp"

#设置要比较的变量名

var_name = "age"

#设置该变量名的类别为：continue_type(连续型变量)；bi_type(二分类型变量)

var_type = "continue_type"

#计算

#com_type:btw_datasets(训练集和验证集之间比较);btw_grp(训练集内部阴性/阳性结局组之间比较,仅适用二分类变量结局)

#training_dataset:训练集数据

#validation_dataset:验证集数据

#var_name:比较的变量名称

#vartype:比较的变量类型：continue_type(连续型变量)；bi_type(二分类型变量)

comparison_nomogram(com_type=com_type,

training_dataset=training_dataset,

validation_dataset=validation_dataset,

var_name=var_name,

var_type=var_type)

################ 3.在训练集中进行单因素和多因素Logistic回归分析 ##########################

##3.1单因素Logistic回归分析

#这里需要你一个个变量进行分析，在下方设置 函数：Death ~ Age。其中，Death为二分类结局的变量名，Age为要进行单因素分析的变量名。

#请根据你自己的研究修改此处的函数

f_lrm <-lrm(Group ~ Age, data=training_dataset, x=TRUE, y=TRUE,maxit=1000)

#查看单因素Logistic分析结果，最下方可见其对应的Coef、p值

print(f_lrm)

##3.2多因素Logistic回归分析

#在上方单因素完成后，根据单因素分析结果，结合研究背景，选择要纳入多因素回归的变量，并修改此处函数(用+号连接自变量)

f_lrm <-lrm(MAKE30 ~ sofa, data=training_dataset, x=TRUE, y=TRUE,maxit=1000)

#查看多因素Logistic分析结果，最下方可见其对应的Coef、p值

print(f_lrm)

################ 4.基于多因素Logistic回归分析结果构建列线图 ##########################

ddist <- datadist(training_dataset)

options(datadist='ddist')

#nomogram计算部分，此处的f_lrm及对应的多因素logistic回归函数。

pdf(file=paste(output_dir, "\\nomogram.pdf", sep = ""),width=10,height=8)

nomogram <- nomogram(f_lrm,fun=function(x)1/(1+exp(-x)), ##逻辑回归计算公式

fun.at = c(0.01,0.1,0.3,0.5,0.7,0.9,0.99),#风险轴刻度

funlabel = "Prob of S-AKI ?", #风险轴便签

lp=F, ##是否显示系数轴

conf.int = F, ##每个得分的置信度区间，用横线表示,横线越长置信度越

abbrev = F#是否用简称代表因子变量

)

#绘制nomogram

plot(nomogram)

dev.off()

################ 5.评估模型（ROC及校准图） ##########################

##5.1训练集中的ROC

pred_f_training<-predict(f_lrm,training_dataset)

#下方参数中Death需改为你的研究的结局变量名

modelroc <- roc(training_dataset$MAKE30,pred_f_training)

#绘制ROC

pdf(file=paste(output_dir, "\\ROC_training.pdf", sep = ""),width=10,height=10)

plot(modelroc, print.auc=TRUE, auc.polygon=TRUE, grid=c(0.1, 0.2),

print.thres=TRUE)

dev.off()

##5.2验证集中的ROC

pred_f_validation<-predict(f_lrm,validation_dataset)

#下方参数中Death需改为你的研究的结局变量名

modelroc <- roc(validation_dataset$MAKE30,pred_f_validation)

#绘制ROC

pdf(file=paste(output_dir, "\\ROC_testing.pdf", sep = ""),width=10,height=10)

plot(modelroc, print.auc=TRUE, auc.polygon=TRUE, grid=c(0.1, 0.2),

print.thres=TRUE)

dev.off()

##5.3训练集中的校准图

cal <- calibrate(f_lrm)

pdf(file=paste(output_dir, "\\calibrate_training.pdf", sep = ""),width=10,height=10)

plot(cal)

dev.off()

##5.4验证集中的校准图

fit.vad<-lrm(validation_dataset$Group~pred_f_validation,data=validation_dataset,x=T,y=T)

pdf(file=paste(output_dir, "\\calibrate_testing.pdf", sep = ""),width=10,height=10)

cal <- calibrate(fit.vad)

plot(cal)

dev.off()

#训练集决策曲线DCA

pdf(file=paste(output_dir, "\\DCA_training.pdf", sep = ""),width=10,height=10)

DCA_training<- decision_curve(Group ~ APTT+Albumin_globulin+BUN+PCT+PLT,data = training_dataset

#,policy = "opt-in"

,study.design = 'cohort')

plot_decision_curve(DCA_training,curve.names= c('Nomogram model'))

dev.off()

#验证集决策曲线DCA

pdf(file=paste(output_dir, "\\DCA_testing.pdf", sep = ""),width=10,height=10)

DCA_training<- decision_curve(Group ~ APTT+Albumin_globulin+BUN+PCT+PLT,data = validation_dataset

#,policy = "opt-in"

,study.design = 'cohort')

plot_decision_curve(DCA_training,curve.names= c('Nomogram model'))

dev.off()
