# Supplementary material for: A risk prediction model based on immune-inflammatory-nutritional indicators for predicting 28-day mortality in sepsis patients with acute respiratory distress syndrome
Source: Front Nutr. 2026 Feb 25;13:1764044. doi: 10.3389/fnut.2026.1764044 (PMC12976859; doi:10.3389/fnut.2026.1764044)
Supplement: Supplementary file 1 [file Data_Sheet_1.zip › Supplementary File 2 - English Version.docx]

################################################################

#### Welcome to the "Medical Nomogram Platform" WeChat Mini Program for using nomograms in clinical practice! ####

#### Also, welcome to follow the "Medical Nomogram" WeChat Official Account ####

#### We regularly share various nomogram tutorials and high-quality nomogram research articles! ####

#### Welcome to follow us and create nomograms together! ####

#### Authors: Team of Professor Chiyuan Ma ####

################################################################

# For instructions on using this code, we will soon release video tutorial courses.

# Welcome to continue following the "Medical Nomogram" WeChat Official Account for updates and resources.

# We have also developed the "Medical Nomogram" WeChat Mini Program for online use of included nomograms.

# For any questions, please contact nomogramhelp@126.com

################################################################

#### The following is the nomogram analysis code for binary outcome variables ####

#### It includes the following main steps: ####

#### 1. Comparison between training and validation datasets ####

#### 2. Comparison between positive and negative outcome groups within the training set ####

#### 3. Univariate and multivariate logistic regression analysis in the training set ####

#### 4. Construction of a nomogram based on multivariate logistic regression results ####

#### 5. Model evaluation (ROC and calibration plots) ####

################################################################

# Tips: 1. Avoid using Chinese characters and spaces in file paths and names. Use underscores _ instead of spaces to separate words.

# 2. Warnings in error messages are acceptable; the code can continue running. If an error occurs, stop and investigate the cause. Continuing blindly will not solve the problem.

################ 0. Environment Preparation and Data Input ##########################

## 0.0 Install required R packages

# (Run this section only for the first use. No need to repeat after successful installation)

install.packages('car')

install.packages('rms')

install.packages('pROC')

# DecisionCurve package is for drawing decision curves and may be difficult to install. Try the following methods:

# 1

install.packages('DecisionCurve')

# 2

BiocManager::install("DecisionCurve", ask = F, update = F)

# 3

library(devtools)

install_github("mdbrown/DecisionCurve")

# 4 This method installs version 1.3

data_dir <- choose.dir(default = "D:\\data_dir", caption = "Select the folder directory where DecisionCurve_1.3.tar.gz is stored")

install.packages(paste0(data_dir, "\\DecisionCurve_1.3.tar.gz"),

repos = NULL,

type = "source")

## 0.1 Load R packages

# Clear all data in R (reset)

rm(list = ls())

library(car)

library(rms)

library(pROC)

library(rmda)

## 0.2 Set the directory for data storage

# Note: Use double backslashes \\ for folder paths

data_dir <- choose.dir(default = "D:\\data_dir", caption = "Select the folder directory for data storage")

## 0.3 Set the directory for exporting images

# Note: Use double backslashes \\ for folder paths

output_dir <- choose.dir(default = "D:\\output_dir", caption = "Select the folder directory for exporting images")

## 0.4 Select the training dataset file (template: logistic_training_dataset.txt)

# Data file format requirements: Save the data file as a txt version.

# Data format requirements: 1. Avoid Chinese characters in the data;

# 2. The first row of the data should contain variable names;

# 3. The first column should be the binary outcome indicator (must be in 0/1 format);

# 4. Other variables should start from the second column;

# 5. Keep variable names consistent between training and validation sets;

# 6. Fill missing values with NA; represent binary variables as 0/1; represent multi-category variables as dummy variables (0/1).

# Template: Generated simulated data.

# Select file path

training_dataset_path <- choose.files(default = data_dir, caption = "Select the txt file for the training dataset.",

multi = TRUE, filters = Filters,

index = nrow(Filters))

# Read data

training_dataset <- read.csv(training_dataset_path, header = TRUE, sep = "\t", stringsAsFactors = FALSE)

# View data

print(paste0("The training set has ", dim(training_dataset)[1], " samples and ", dim(training_dataset)[2], " variables."))

## 0.5 Select the validation dataset file (template: logistic_validation_dataset.txt)

# Data file format requirements: Save the data file as a txt version.

# Data format requirements: 1. Avoid Chinese characters in the data;

# 2. The first row of the data should contain variable names;

# 3. The first column should be the binary outcome indicator (must be in 0/1 format);

# 4. Other variables should start from the second column;

# 5. Keep variable names consistent between training and validation sets;

# 6. Fill missing values with NA; represent binary variables as 0/1; represent multi-category variables as dummy variables (0/1).

# Template: Generated simulated data.

# Select file path

validation_dataset_path <- choose.files(default = data_dir, caption = "Select the txt file for the validation dataset.",

multi = TRUE, filters = Filters,

index = nrow(Filters))

# Read data

validation_dataset <- read.csv(validation_dataset_path, header = TRUE, sep = "\t", stringsAsFactors = FALSE)

# View data

print(paste0("The validation set has ", dim(validation_dataset)[1], " samples and ", dim(validation_dataset)[2], " variables."))

################ 1. Comparison Between Training and Validation Datasets ##########################

com_type <- "btw_datasets"

var_name <- "Gender"

# Define the comparison function (run this definition directly without modification)

comparison_nomogram <- function(com_type, training_dataset, validation_dataset, var_name, var_type) {

# com_type: btw_datasets (comparison between training and validation sets); btw_grp (comparison between negative/positive outcome groups within training set, only for binary outcome variables)

# training_dataset: training dataset

# validation_dataset: validation dataset

# var_name: variable name to compare

# var_type: variable type: continue_type (continuous variable); bi_type (binary variable)

# Extract data

if (com_type == "btw_datasets") {

data_1 <- training_dataset[, var_name]

data_2 <- validation_dataset[, var_name]

} else if (com_type == "btw_grp") {

data_1 <- training_dataset[(training_dataset[, 1] == 0), var_name]

data_2 <- training_dataset[(training_dataset[, 1] == 1), var_name]

}

# Calculate data

if (var_type == "continue_type") {

judge_p <- function(p1, p2, p3, tp, zhp) {

mark <- 0

if (p1 < 0.05) {

mark <- 1

}

if (p2 < 0.05) {

mark <- 1

}

if (p3 < 0.05) {

mark <- 1

}

if (mark == 0) {

return(tp)

} else {

return(zhp)

}

}

grp_1_ztp <- shapiro.test(data_1)[2][[1]]

grp_2_ztp <- shapiro.test(data_2)[2][[1]]

y_leveneT <- c(data_1, data_2)

group_leveneT <- as.factor(c(rep(1, length(data_1)), rep(2, length(data_2))))

fcp <- leveneTest(y_leveneT, group_leveneT)[3][[1]][1]

t_testp <- t.test(data_1, data_2, paired = F)[3][[1]]

df <- data.frame(y_leveneT, group_leveneT)

zhp <- wilcox.test(y_leveneT ~ group_leveneT, df)[3][[1]]

finalp <- judge_p(p1 = grp_1_ztp, p2 = grp_2_ztp, p3 = fcp, tp = t_testp, zhp = zhp)

} else if (var_type == "bi_type") {

# Related functions

col_matrix_producer <- function(grp_0_dingxing_data, grp_1_dingxing_data) {

vars <- unique(c(names(table(grp_0_dingxing_data)), names(table(grp_1_dingxing_data))))

col_matrix <- matrix(data = 0, nrow = length(vars), ncol = 2, byrow = FALSE, dimnames = NULL)

colnames(col_matrix) <- c("grp_0", "grp_1")

rownames(col_matrix) <- vars

for (index_var in 1:length(vars)) {

if (!is.na(table(grp_0_dingxing_data)[vars[index_var]][[1]])) {

col_matrix[vars[index_var], "grp_0"] <- table(grp_0_dingxing_data)[vars[index_var]][[1]]

}

}

for (index_var in 1:length(vars)) {

if (!is.na(table(grp_1_dingxing_data)[vars[index_var]][[1]])) {

col_matrix[vars[index_var], "grp_1"] <- table(grp_1_dingxing_data)[vars[index_var]][[1]]

}

}

return(col_matrix)

}

col_matrix_ratio_producer <- function(col_matrix, round_num) {

out_table <- col_matrix

for (index_col in 1:ncol(col_matrix)) {

sum_col <- sum(col_matrix[, index_col])

for (index_row in 1:nrow(col_matrix)) {

ratio <- round(100 * col_matrix[index_row, index_col] / sum_col, round_num)

out_table[index_row, index_col] <- paste0(col_matrix[index_row, index_col], " (", ratio, "%)")

}

}

return(out_table)

}

## Remove possible missing values

b0 <- which(data_1 == "NA")

if (length(b0)) {

data_1 <- data_1[-b0]

}

b1 <- which(data_2 == "NA")

if (length(b1)) {

data_2 <- data_2[-b1]

}

# Generate contingency table

col_matrix <- col_matrix_producer(data_1, data_2)

# Calculate results

chisqcp <- chisq.test(col_matrix)[3][[1]]

fisherp <- tryCatch(fisher.test(col_matrix)[1][[1]], error = function(e) { return("A") })

if (fisherp == "A") {

fisherp <- fisher.test(col_matrix, simulate.p.value = TRUE)[1][[1]]

}

col_matrix_observed <- chisq.test(col_matrix)$observed

col_matrix_expected <- chisq.test(col_matrix)$expected

round_num <- 1

col_matrix_ratio <- col_matrix_ratio_producer(col_matrix, round_num)

# Total observations

sum_N <- sum(col_matrix)

# Decide between chi-square and Fisher's test

if (any(col_matrix_expected < 5) | sum_N < 40) {

finalp <- fisherp

} else {

finalp <- chisqcp

}

}

return(paste0("The p-value for variable ", var_name, " is: ", finalp))

}

# Since different data types require different statistical methods, you need to compare variables one by one and set the variable category each time.

# Set whether to compare between training and validation sets (btw_datasets) or between negative/positive outcome groups within the training set (btw_grp, only for binary outcome variables)

com_type <- "btw_datasets"

# Set the variable name to compare

var_name <- "age"

# Set the variable type: continue_type (continuous variable); bi_type (binary variable)

var_type <- "continue_type"

# Calculate

comparison_nomogram(com_type = com_type,

training_dataset = training_dataset,

validation_dataset = validation_dataset,

var_name = var_name,

var_type = var_type)

################ 2. Comparison Between Positive and Negative Outcome Groups Within the Training Set ##########################

# Set whether to compare between training and validation sets (btw_datasets) or between negative/positive outcome groups within the training set (btw_grp, only for binary outcome variables)

com_type <- "btw_grp"

# Set the variable name to compare

var_name <- "age"

# Set the variable type: continue_type (continuous variable); bi_type (binary variable)

var_type <- "continue_type"

# Calculate

comparison_nomogram(com_type = com_type,

training_dataset = training_dataset,

validation_dataset = validation_dataset,

var_name = var_name,

var_type = var_type)

################ 3. Univariate and Multivariate Logistic Regression Analysis in the Training Set ##########################

## 3.1 Univariate Logistic Regression Analysis

# Here, you need to analyze variables one by one. Set the formula below: Death ~ Age.

# Replace "Death" with your binary outcome variable name and "Age" with the variable name for univariate analysis.

# Modify the formula according to your study.

f_lrm <- lrm(Group ~ Age, data = training_dataset, x = TRUE, y = TRUE, maxit = 1000)

# View univariate logistic analysis results. Coefficients and p-values are shown at the bottom.

print(f_lrm)

## 3.2 Multivariate Logistic Regression Analysis

# After univariate analysis, select variables for multivariate regression based on univariate results and research background. Modify the formula below (connect independent variables with +).

f_lrm <- lrm(MAKE30 ~ sofa, data = training_dataset, x = TRUE, y = TRUE, maxit = 1000)

# View multivariate logistic analysis results. Coefficients and p-values are shown at the bottom.

print(f_lrm)

################ 4. Construct Nomogram Based on Multivariate Logistic Regression Results ##########################

ddist <- datadist(training_dataset)

options(datadist = 'ddist')

# Nomogram calculation part. Use the f_lrm object and the corresponding multivariate logistic regression formula.

pdf(file = paste(output_dir, "\\nomogram.pdf", sep = ""), width = 10, height = 8)

nomogram <- nomogram(f_lrm, fun = function(x) 1 / (1 + exp(-x)), ## Logistic regression calculation formula

fun.at = c(0.01, 0.1, 0.3, 0.5, 0.7, 0.9, 0.99), # Risk axis ticks

funlabel = "Prob of S-AKI ?", # Risk axis label

lp = F, ## Whether to display the coefficient axis

conf.int = F, ## Confidence intervals for each score, represented by horizontal lines; longer lines indicate higher confidence

abbrev = F # Whether to use abbreviations for factor variables

)

# Plot nomogram

plot(nomogram)

dev.off()

################ 5. Model Evaluation (ROC and Calibration Plots) ##########################

## 5.1 ROC in the training set

pred_f_training <- predict(f_lrm, training_dataset)

# Replace "Death" with your outcome variable name in the parameters below

modelroc <- roc(training_dataset$MAKE30, pred_f_training)

# Plot ROC

pdf(file = paste(output_dir, "\\ROC_training.pdf", sep = ""), width = 10, height = 10)

plot(modelroc, print.auc = TRUE, auc.polygon = TRUE, grid = c(0.1, 0.2),

print.thres = TRUE)

dev.off()

## 5.2 ROC in the validation set

pred_f_validation <- predict(f_lrm, validation_dataset)

# Replace "Death" with your outcome variable name in the parameters below

modelroc <- roc(validation_dataset$MAKE30, pred_f_validation)

# Plot ROC

pdf(file = paste(output_dir, "\\ROC_testing.pdf", sep = ""), width = 10, height = 10)

plot(modelroc, print.auc = TRUE, auc.polygon = TRUE, grid = c(0.1, 0.2),

print.thres = TRUE)

dev.off()

## 5.3 Calibration plot in the training set

cal <- calibrate(f_lrm)

pdf(file = paste(output_dir, "\\calibrate_training.pdf", sep = ""), width = 10, height = 10)

plot(cal)

dev.off()

## 5.4 Calibration plot in the validation set

fit.vad <- lrm(validation_dataset$Group ~ pred_f_validation, data = validation_dataset, x = T, y = T)

pdf(file = paste(output_dir, "\\calibrate_testing.pdf", sep = ""), width = 10, height = 10)

cal <- calibrate(fit.vad)

plot(cal)

dev.off()

# Decision curve analysis (DCA) in the training set

pdf(file = paste(output_dir, "\\DCA_training.pdf", sep = ""), width = 10, height = 10)

DCA_training <- decision_curve(Group ~ APTT + Albumin_globulin + BUN + PCT + PLT, data = training_dataset

#, policy = "opt-in"

, study.design = 'cohort')

plot_decision_curve(DCA_training, curve.names = c('Nomogram model'))

dev.off()

# Decision curve analysis (DCA) in the validation set

pdf(file = paste(output_dir, "\\DCA_testing.pdf", sep = ""), width = 10, height = 10)

DCA_training <- decision_curve(Group ~ APTT + Albumin_globulin + BUN + PCT + PLT, data = validation_dataset

#, policy = "opt-in"

, study.design = 'cohort')

plot_decision_curve(DCA_training, curve.names = c('Nomogram model'))

dev.off()
